# Characterization of Proliferating Neural Progenitors after Spinal Cord Injury in Adult Zebrafish

**DOI:** 10.1371/journal.pone.0143595

**Published:** 2015-12-02

**Authors:** Subhra Prakash Hui, Tapas Chandra Nag, Sukla Ghosh

**Affiliations:** 1 Department of Biophysics, Molecular Biology and Bioinformatics, University of Calcutta, 92, A. P. C. Road, Kolkata—700009, India; 2 Department of Anatomy, All India Institute of Medical Sciences, New Delhi- 110029, India; Wayne State University School of Medicine, UNITED STATES

## Abstract

Zebrafish can repair their injured brain and spinal cord after injury unlike adult mammalian central nervous system. Any injury to zebrafish spinal cord would lead to increased proliferation and neurogenesis. There are presences of proliferating progenitors from which both neuronal and glial loss can be reversed by appropriately generating new neurons and glia. We have demonstrated the presence of multiple progenitors, which are different types of proliferating populations like Sox2^+^ neural progenitor, A2B5^+^ astrocyte/ glial progenitor, NG2^+^ oligodendrocyte progenitor, radial glia and Schwann cell like progenitor. We analyzed the expression levels of two common markers of dedifferentiation like *msx-b* and vimentin during regeneration along with some of the pluripotency associated factors to explore the possible role of these two processes. Among the several key factors related to pluripotency, *pou5f1* and *sox2* are upregulated during regeneration and associated with activation of neural progenitor cells. Uncovering the molecular mechanism for endogenous regeneration of adult zebrafish spinal cord would give us more clues on important targets for future therapeutic approach in mammalian spinal cord repair and regeneration.

## Introduction

Unlike fish and urodele amphibians which can regenerate their CNS in adult life, the adult mammalian central nervous system (CNS) shows rather limited capacity to regenerate after injury. Any spinal cord that undergoes successful regeneration would require rapid growth and proliferation leading to neurogenesis and axonogenesis. Moreover, injury induced tissue loss after CNS injury would require replenishment of lost cells both by neurogenesis and gliogenesis.

Neurogenesis in adult mammals is tightly restricted to the subependymal zone (SEZ) of the lateral wall of the ventricle and the subgranular zone (SGZ) of the hippocampus but appear to be more widespread in other vertebrates like reptiles [1], birds [2], and fish [3]. The evidence of neurogenesis in adult mammalian forebrain also raises the issue of presence of neural stem cells (NSCs) in CNS [4]. In teleost fish, proliferation and neurogenesis occur throughout their life, correlating with long lasting brain and spinal cord growth and a high capacity for regeneration [3,5–8]. In the adult zebrafish brain, it has already been reported that different neuronal subtypes can be generated from different parts of brain other than olfactory bulb (OB) or hippocampal granule interneurons [5,9]. Further studies involving this model might throw light into the mechanism(s) of generating different neuronal subtypes in regenerating cord similar to other parts of CNS. Earlier studies on zebrafish have also suggested that proliferation and neurogenesis occur in different areas of brain and spinal cord [5,6,9–14]. The proliferating progenitors in adult zebrafish brain and retina had been shown to have retained stem cell like properties, similar to what has been observed in mammalian CNS [10,15]. Both neurons and glias can be derived from adult neural progenitor as reported in teleost hind brain [16].

Discovery of stem cell in adult mammalian CNS led to the possibility of stimulating endogenous progenitor population. This could be targeted for therapeutic purpose to induce regeneration after any spinal cord injury (SCI). The objective of our study is to identify and characterize progenitor(s) that may contribute to different neural population in the regenerating cord. The present study demonstrates the presence of proliferating cells in the spinal cord of adult zebrafish when inflicted with crush injury and that may function as neural progenitor cells. There are multiple progenitors in a regenerating cord and these cells are localized not only around the ependyma, like radial glia but are also in white matter (WM). Different types of progenitors such as neuronal, glial- astrocytes/oligodendrocyte as well as Schwann cell progenitors have been identified by expression of several markers, i.e., HuC/D, Sox2, OCT4, A2B5 (GQ1c ganglioside), NG2 (nerve-glial antigen 2), GFAP (glial fibrillary acidic protein), BLBP (brain lipid binding protein), GLAST (glutamate astrocyte-specific transporter), MAG (myelin associated glycoprotein), CNPase (2', 3'-cyclic nucleotide 3'-phosphodiesterase) and by employing ultra-structural analysis.

The non-radial glial neuronal precursors may also contribute to the process of neurogenesis since it has been documented that neurogenic region of adult mammalian brain expresses Sox2. The Sox2 expression is present in proliferating precursors and in glial cells that are believed to represent stem cells [17]. Similarly, Sox2 expression has been demonstrated in telencephalic ventricular region of adult zebrafish brain [5] and spinal cord [18,19]. Here we confirm the presence of Sox2 positive proliferating precursors in regenerating spinal cord along with other progenitors.

There are ample evidences to suggest that regeneration of appendages in urodeles and fish involve dedifferentiation of terminally differentiated cells, namely, keratinocytes, fibroblasts, osteoblast and myotubes [20–23]. Following an extensive proliferation, blastema would redifferentiate to yield all different cell types to generate a faithful copy of the missing structure [24–26]. Recent discovery of reprogramming of mammalian somatic cell to achieve pluripotency [27,28] has rekindled our interest to understand the molecular mechanism that could promote reprogramming or maintenance of stem cell like population in complex regenerating organs. Reversal of differentiated state is the common event during organ regeneration [29] and in order to uncover the mechanism(s) associated with these two processes, we show expression of several markers associated with dedifferentiation and reprogramming during CNS regeneration in zebrafish.

## Materials and Methods

This study was carried out in accordance with the recommendation in the guidelines provided by CPCSEA (Committee for the Purpose of Control and Supervision of Experiments on Animals, Ministry of Environments and Forests, Government of India). The protocol (Part B), which includes all the details of surgical processes inflicted and anesthesia requirement for injuring and sacrificing animals, was approved by the Institutional Animal Ethics committee, Department of Biophysics, Molecular Biology and Bioinformatics, University of Calcutta under registration with CPCSEA (CPCSEA/ORG/CH/Reg No. 925/295).

### Fish maintenance and surgical procedures

Zebrafish stock populations were either obtained from local pet shop or bred in our animal house facility. Fishes were kept in separate groups of 15–20 in the aquatic system maintained at 28°C on a 14 hr light/ 10 hr dark cycle. Three to six month old fishes with same male and female ratio were subjected to spinal cord injury. Fishes were anaesthetized with 0.02% tricaine (MS222; Sigma), and their spinal cord was exposed at the level of dorsal fin. The spinal cords were given crush injury, as described previously [6]. The animals were then allowed to regenerate at 28°C until the end of the experiment.

### BrdU incorporation

BrdU (50μl of 2.5mg/ml; Bromo-deoxy-Uridine, Sigma-Aldrich, USA) was injected intraperitoneally, 24 hr before the collection of tissues at different time points like 3 dpi, 7 dpi and 15 dpi. Spinal cord tissues equivalent of approximately 2 mm length was collected from both injured and uninjured animals for further processing.

### Immunohistochemistry and transmission electron microscopy (TEM)

Immunostaining was performed as reported in an earlier communication [6] by using primary antibodies shown to specifically recognize fish, amphibian and mammalian proteins. Cross reactivity of CNPase, MAG, OCT-4 and NG2 were tested by running Western blot and results are shown in S5 Fig. Alignment of OCT-4 protein sequences for zebrafish and human revealed an identity of 70% using the NCBI Protein-Protein BLAST network service. OCT-4 antibody is directed against synthetic peptide (AA282-297 of human OCT-4, according to manufacturer’s information, Millipore, AB3209). The peptide domain of OCT-4 has more than 80% similarity with *Danio* Oct-4. Also, high similarities in amino acid alignment of *Danio* siglec-4 (alternative name MAG) with mammalian representatives of siglec-4 and MAG also showed cross reactivity with other species like axolotl [30]. A2B5 is specific for ganglioside GQ1c from multiple species [31,32] and in zebrafish stains appropriate progenitor cells as mentioned in literature [33]. List of other antibodies include anti-GFAP (1:500, DAKO, USA, Cat. no. 20334) [5,13,34]; anti-BrdU (1:300, Sigma, USA, Cat. no. B-8434) [6,18]; anti-HuC/D (1:50, Molecular Probes, USA, Cat. no. A-21271) [11,18]; anti-Sox2 (1:400, Abcam, USA, Cat. no. Ab15830) [11,35,36]; anti-A2B5 (1:250, Millipore, USA, Cat. no. MAB312R) [37]; anti-NG2 (1:100, Millipore, USA, Cat. no. AB5320) [38]; anti-CNPase (1:200, Millipore, USA, Cat. no. MAB326R) [39,40]; anti-MAG (1:100, Santa Cruz Biotechnology, USA, Cat. no. SC-15324) [30]; anti-BLBP (1:400, Abcam, USA, Cat. no. Ab27171) [9,10,41]; anti-GLAST (1:400, Chemicon, USA, Cat. no. AB1782) [9, 10,41]; OCT4 (1:200, Millipore, USA, Cat. no. AB3209) [42]; Vimentin (1:300, Millipore, USA, Cat. no. MAB3400) [35,43–46]; PCNA (1:100, Santa Cruz Biotechnology, USA, Cat. no. SC-7907) [47]. Briefly, sections were rehydrated and given several wash in phosphate buffered saline (PBS) with 0.1% Tween-20 or Triton X-100 (PBST). Then sections were incubated with blocking solution for 1hr (5% goat/rabbit/donkey serum, 1% BSA in PBS) and then with primary antibody for either 1hr at room temperature or overnight at 4°C. All the primary antibodies were diluted in PBS. To unmask some antigens in paraformaldehyde fixed tissue, antigen retrieval has been done by keeping the slides in 95°C water bath for 15 min either in sodium-citrate buffer (pH 6.0) or in Tris-buffer (pH 8.0) before incubation with the primary antibody. The following secondary antibodies were used: Rhodamine-conjugated Goat anti-rabbit antibody (1:200, Santa Cruz Biotechnology, USA); Rhodamine-conjugated donkey anti-goat antibody (1:200, Santa Cruz Biotechnoloy, USA); TRITC-conjugated goat anti-rabbit IgG antibody (1:400, Jackson ImmunoResearch Laboratories, USA); FITC-conjugated goat anti-rabbit IgG antibody (1:400, Jackson ImmunoResearch Laboratories, USA); FITC-conjugated goat anti-mouse (1:200, Santa Cruz Biotechnoloy, USA); TRITC-conjugated goat anti-mouse (1:400, Jackson ImmunoResearch Laboratories, USA). Nuclei were counter-stained either with DAPI (1:1000, Sigma, USA). BrdU immunohistochemistry on uninjured and injured spinal cord was done according to [6].

For TEM analysis spinal cord tissues were also fixed, sectioned and observed for TEM as described in [6]. Application of Fiji software was used to measure the nuclear and cytoplasmic lengths for identifying progenitor cells from TEM images.

### Microscopy and cell quantification

Immunostained tissue sections were photographed by using an Olympus fluorescent microscope (model; BX 51) with ImagePro Express software. In all colocalization study, the quantification of cell was done from all optical images obtained by using a Zeiss LSM 510 Meta (inverted) confocal microscope. Six representative sections from 1 mm uninjured or injured from each spinal cord were averaged to compute percentage of particular cells and colocalized cells for each animal. In case of injured cord three representative sections from 500 μm both rostral and caudal side of injury epicenter were averaged for quantification. Cell counting was carried out manually using a cell counter application of ImageJ software. During the quantification process, cells were considered positive if cells were found stained with specific markers and also colocalized with DAPI. The colocalized cells and proliferating cells were considered positive only when both markers showed clear overlapping and cell nucleus overlapping with BrdU respectively. For quantifying NG2^+^ cells only bipolar single nucleated cells were considered positive as possible oligodendrocyte progenitors. Long, tubular, multi-nucleated cells were excluded in this study.

### Immunoblotting

Zebrafish spinal cord was collected 7 and 10 days after injury along with the control, uninjured cords. Tissues were prepared in extraction buffer (37.5 mM Tris, 75 mM Nacl, 0.5% Triton X-100, protease inhibitor cocktail) and then the resulting tissue lysates were subjected to either 7.5% or 10% sodium dodecyl sulfate polyacrylamide gel electrophoresis (SDS-PAGE). After the electrophoresis, proteins were transferred onto nitrocellulose membranes and subjected to western blotting. The western blots were developed with antibody anti-CNPase (1:1000, Millipore, USA), anti- MAG (1:100, Santa Cruz Biotechnology, USA), anti- OCT4 (1:200, Millipore, USA) and anti-NG2 (1:500, Millipore, USA) followed by anti-mouse or anti-rabbit alkaline phosphatase coupled secondary antibody (1:1,000 Jackson laboratory, USA). GAPDH was used as internal loading control protein. Protein bands were visualized using NBT/BCIP as substrate. PageRuler Broad Range Protein Ladder (Thermo Scientific, USA) was used as a standard molecular weight marker.

### Enzyme linked immunosorbant assay (ELISA)

Analysis of Msx-1 protein by using ELISA assay was done according to [6]. Briefly, tissue extract of spinal cord tissue samples (65ug protein /well) were added to the wells of ELISA plate and incubated overnight at 4°C. Subsequently blocking buffer (1% BSA in PBS) containing primary antibody, anti-Msx1 (1:100, Santa Cruz Biotech, USA) were added and incubated for 1 hr at 37°C. After incubation with primary antibody, wells were washed thrice in washing buffer (0.5% BSA, 0.5% NE-40 in PBS) followed by incubation with secondary antibody (HRP conjugated anti-rabbit antibody (1:1000 dilutions) for 1 hr at 37°C. Several washes were given before adding the substrate TMB (tetramethyl benzidine). Color reaction was developed in dark and stopped by addition of 1M H_2_SO_4_. The optical density was measured at 450nm by using an ELISA reader (Bio RAD, USA). **Quantitative real-Time RT-PCR (qRT-PCR):** RNA was prepared from 3 biological replicates. For each biological replicate, spinal cord from about 15–20 fishes were pooled in both the control and in the regenerating samples. Control fishes and fishes possessing injury in the spinal cord were anesthetized deeply for 5 minutes in 0.1% tricaine (MS222; Sigma, USA) and approximately 1mm length of spinal cord from injury epicenter were dissected out (both rostral and caudal) from 15–20 fishes in each batch and pooled for RNA extraction. The extraction of RNA and qRT-PCR was done following the method mentioned in Hui et al., 2014. The specific gene primers like zebrafish *sox2* (F 5’ AGAGTTTCTCACTGAAGGTACATAG 3’ and R 5’ TGCCTCTGTTCGTTCTCTCA 3’), *pou5f1* (F 5’ AACTCCGAGAACCCTCAGGA 3’ and R 5’ TCGTTTTCTTTTTCGCGTGTCG 3’), *msx1-b/msxB* (F 5’ GAACAGAGCACTTGGTCAAACTC 3’ and R 5’ TCCTGTTCGTCTTGTGCTTGC 3’, *msx-c* (F 5’ AGTGACAAGGGACAGTCCGGCT 3’ and R 5’ TAAACGGGGTCCGCGGTTTTCG 3’), *msx1-a*/*msxe* (F 5’ TTCTGCAGCTGCCGGTGAAG 3’ and R 5’ TTTTCTCAGAGGGCACGCGG 3’) the internal control gene *β-actin1* (F 5’ GCTCACCATGGATGATGATATCGC 3’ and R 5’ GGAGGAGCAATGATCTTGATCTTC 3’) were amplified in separate reaction tubes. No primer -dimers were obtained for either the target genes or beta-actin as assessed by melt curve analysis. The specificity of the products was also confirmed by melt curve analysis. The PCR cycles in all cases were started with Taq activation at 94°C for 5 mins and followed by final extension of 72ºC for 7 mins.

### In situ hybridization tissue section

For in situ hybridization, pCRIITOPO vector (Invitrogen) containing *pou5fl1* and *Msx-b* cDNA clones were obtained from Genome Institute of Singapore, zebrafish genome resources. Both sense and antisense RNA probes were generated using a digoxigenin (DIG) RNA labeling kit (Roche Diagnostics, Laval, Que´bec, Canada) following the manufacturer’s instructions. Hybridization of sense and antisense probe were carried out on tissue section as mentioned by [18]. Photographs were taken under light microscope using an Olympus microscope (model; BX 51) and a Leica Microsystem microscope with build camera (DFC 290).

### Statistical analysis

The statistical analyses were performed with Microsoft Excel (Office 2000) and all experimental data have been expressed as Mean ± s.e.m. and numbers of spinal cord samples used are mentioned in the corresponding legends of each figure. The data obtained were compared using unpaired two-tailed Student’s t-test when comparing two groups and one-way ANOVA followed by Bonferroni post-hoc test when comparing more than two groups. The P values were determined using either GraphPad Prism or Statistica software. Error bars represent s.e.m. and statistical significance are represented as ***P≤0.001; **P≤ 0.01; *P≤0.05. P>0.05 was not considered as significant.

## Results

### Presence of proliferating progenitor: Identification of NSC like cells

**Fig 1:**
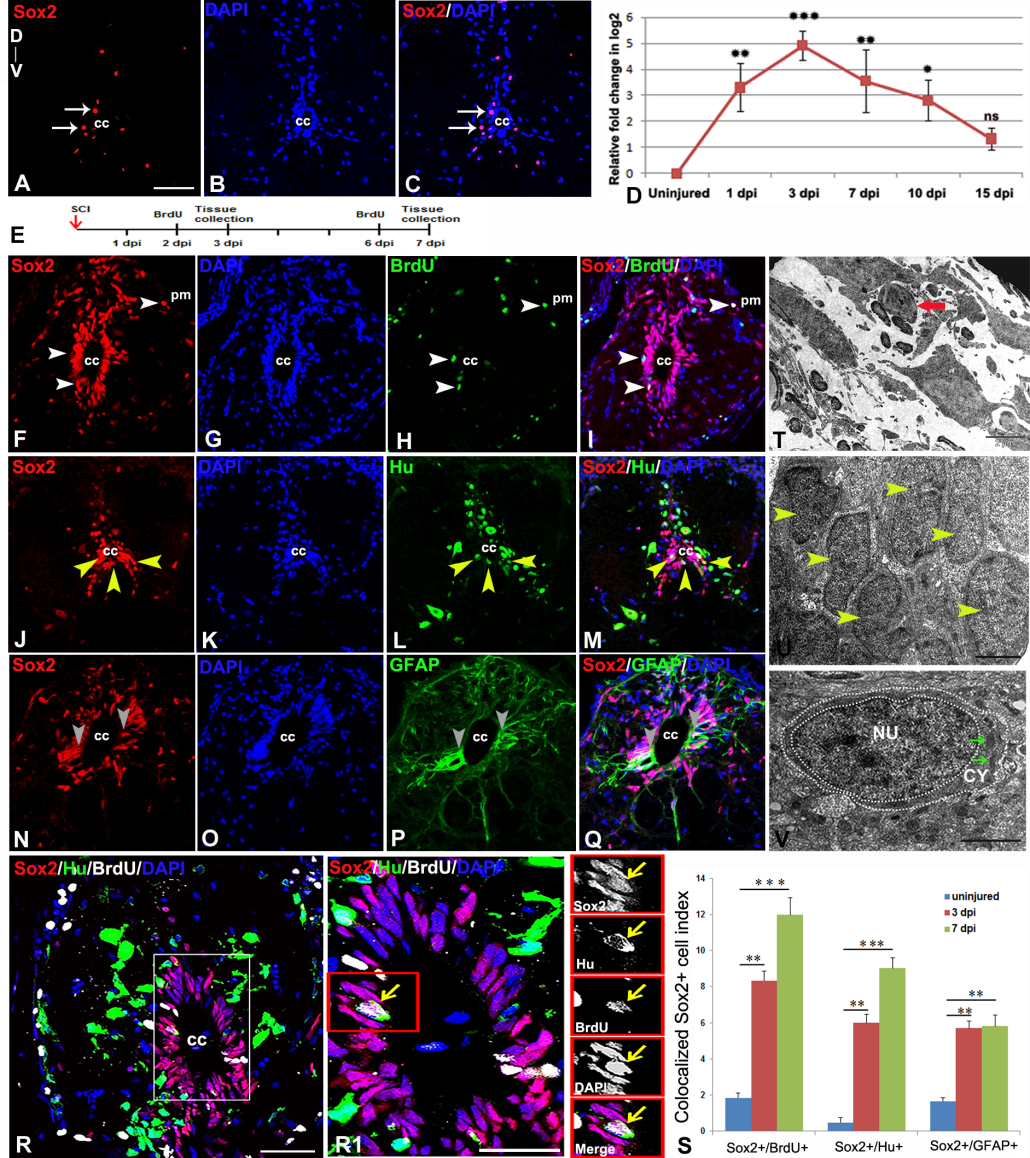
Immunohistochemical, ultrastructural and quantitative analysis of proliferating neural progenitors in injured zebrafish spinal cord **:** A-C) An uninjured cord section showing a few SOX2^+^ (A, arrow) cells in the grey matter around central canal (cc). D) Quantitative RT-PCR of *sox2* expression showing fold change (red graph) and pattern of expression at different time points after injury. Error bar indicates the value of s.e.m. (n = 3) and statistical significance represented as p value (ANOVA; ***P≤0.001, **P≤ 0.01, *P≤0.05, ns = not significant). E) Drawing represents the time frame of SCI, BrdU treatment and tissue collection for cell proliferation experiments. F-I) A 3 dpi cord section shows many SOX2^+^positive cells in ependyma around the central canal (cc) and also near pial membrane (pm), counter stained with DAPI (G) In the same section some of the SOX2^+^ cells are also proliferating (H) and are SOX2^+^/BrdU^+^ (I, white arrowheads). Note that some SOX2^+^/BrdU^+^ colocalized cells are also present near pial membrane (white arrows). J-M) A 3 dpi cord section shows many SOX2^+^ positive cells (J, yellow arrowheads) in ependyma around the central canal (cc), counter stained with DAPI. (K) In same section some of the SOX2^+^ cells are colocalized with HuC/D^+^ cells (M, yellow arrowheads). N-Q) A 3 dpi cord section showing many SOX2^+^ cells (N, grey arrowheads) in the grey matter, counter stained with DAPI (O) and GFAP^+^ cells (P) around ependyma. Same section showing few SOX2^+^/GFAP^+^ colocalized cells (grey arrowheads) around the central canal (cc). R) A 3 dpi cord section stained with SOX2, HuC/D, BrdU and DAPI. R1) Higher magnification of boxed area in R showing a colocalized SOX2^+^/HuC/D^+^/BrdU^+^ cell around central canal (yellow arrow; cc). S) Bar graph indicates quantification of proliferating SOX2^+^ cells, SOX2^+^/HuC/D^+^ colocalized cells and SOX2^+^/GFAP^+^ colocalized cells in uninjured, 3 dpi and 7 dpi cord after crush injury. Values represent as mean ± s.e.m. (n = 5). Statistical significance represented as p value (Student’s t-test; *p<0.01, **p<0.001). T) A small sized progenitor (red arrow) with compact nucleus in the injury epicenter of a 3 dpi cord section. U) Several newly formed cells (yellow arrowheads) in the injury site of a 7 dpi cord with progenitor phenotype. V) A newly formed progenitor cell with few cytoplasmic organelles (green arrows) in the injury site of a 10 dpi cord. ‘NU’ and ‘CY’ denote nucleus and cytoplasm respectively, white dotted line demarcate the boundaries of the nucleus and the cell. The values in bar represents Mean ± s.e.m. (n = 5), statistical significance represented as p value (Student’s t-test; **p<0.01, ***p<0.001). ‘cc’ denotes central canal of the cord. Scale bar = 50 μm (A-C, F-Q), 20 μm (R), 10 μm (R1), 2 μm (T), 1 μm (U), 500 nm (V).

Replacement of tissue due to high proliferative response after SCI in zebrafish was observed by others [13] and also by us [6]. Presence of proliferating progenitor(s) in spinal cord can be demonstrated by transmission electron microscopy (TEM) analysis and immunohistochemical localization of proliferating cell markers. To characterize proliferating progenitors, we have analysed BrdU incorporation in injured cord along with Sox2, a marker for neural progenitor (Fig 1, S1 Fig) as suggested by many authors in different organism, such as zebrafish, axolotl and *Xenopus* [14, 48]. We found that Sox2 is expressed only in few cells of the grey matter in adult uninjured spinal cord (Fig 1A and 1C). But the expression of Sox2 is upregulated in injured spinal cord as shown by time course analysis of *sox2* mRNA and the highest level of expression is observed in 3 dpi cord (Fig 1D). Immunohistological analysis also confirms increased expression of Sox2 at 3 dpi cord and which sustains until 7 dpi, where almost all ependymal cells around the central canal are expressing Sox2 as shown in representative sections taken from injury epicenter of different injured cords (Fig 1F, 1J, 1N and 1R, S1A Fig). Increased proliferation of BrdU incorporation was first observed in 3 dpi cord followed by highest proliferation in 7 dpi and subsequent decrease in proliferation in 10 and 15 dpi cords [6]. A significant increase in Sox2^+^/BrdU^+^ cells were observed in both 3 dpi (p<0.01) and 7 dpi (p<0.001) cords compared to uninjured cord (Fig 1S, S1 Table). Proliferating Sox2^+^ cells are mostly present in the grey matter, particularly in the ventricular zone around the central canal and in subependyma (Fig 1I, 1R and 1R1, S1B Fig). A few cells near pial membrane are also showing colocalization of Sox2 and BrdU (Fig 1I). Increased number of proliferating Sox2^+^ cells in 3 dpi (8.34%, S1 Table) and 7 dpi (11.98%, S1 Table) cords suggest that these cells are proliferating progenitors. Although the number of Sox2 positive cells are more (data not shown) but the colocalized Sox2^+^/BrdU^+^ cells are less in 3 dpi cord compared to 7 dpi cord because the proliferation rate in 3 dpi cord is lower than that of 7 dpi cord. Sox2^+^ cells in grey matter are also colocalized with newly formed neurons as they are expressing HuC/D protein, approximately 6% and 9% Sox2^+^ cells are HuC/D^+^ in 3 day and 7 day post injured cord respectively compared to uninjured cord where less than 0.5% Sox2^+^ cells are HuC/D^+^ (Fig 1M and 1S S1 Table). Colocalization of Sox2 with ependymo-radial glial marker GFAP indicates that 5.69% and 5.81% of the Sox2 positive cells are GFAP^+^ radial glia in 3 day and 7 day injured cord respectively (Fig 1Q and 1S, S1 Table) as confirmed by comparing their morphological characters (Fig 1P and 1Q).

Ultrastructural analysis of injured cord showed existence of progenitor cells both in early stage like 3, 7 dpi cord and in late stage like 10 dpi cord (Fig 1T–1V). Progenitors were characterized by high nuclear-cytoplasmic index (also known as the nucleus-cytoplasm ratio, N:C ratio, or N/C index), a measurement by which a ratio of the size (i.e., volume) of the nucleus of a cell to the size of the cytoplasm of that cell is analysed. The N/C index indicates the maturity of a cell, because as a cell matures the size of its nucleus generally decreases [49,50]. For determining N/C index, the nuclear and cytoplasmic lengths were measured using the application of Fiji software for identifying progenitor cells from TEM images. We observed that at early stage, progenitors have very little cytoplasm but a large nucleus thus showing very high N/C index in 3 and 7 dpi cord (Fig 1T and 1U). The obvious presence of progenitors in late stage at 10 dpi cord (Fig 1V) also showed high N/C index and can be recognized as these cells have dense chromatin pattern in nucleus. Other features are absence of cytoplasmic filaments but appearance of a few cytoplasmic organelles such as rough endoplasmic reticulum and Golgi apparatus [51–53].

### Characterization of glial progenitors: Proliferating astrocyte and oligodendrocyte progenitors after SCI

A2B5 gangliosides are one of the earliest markers for glial restricted precursors (GRP) during mammalian CNS development. These A2B5 expressing cells identify lineage restricted glial precursors, that exist in developing mouse neural tube, among embryonic stem (ES) cells and are similar to rat GRP [54]. These restricted precursors are known to generate oligodendrocyte and astrocytes but not neurons [55,56]. Furthermore, transplantation of GRPs, isolated from fetal spinal cord into injured cord, is capable of differentiating into astrocytic and oligodendrocytic lineages but not to a neuronal lineage [57].

**Fig 2:**
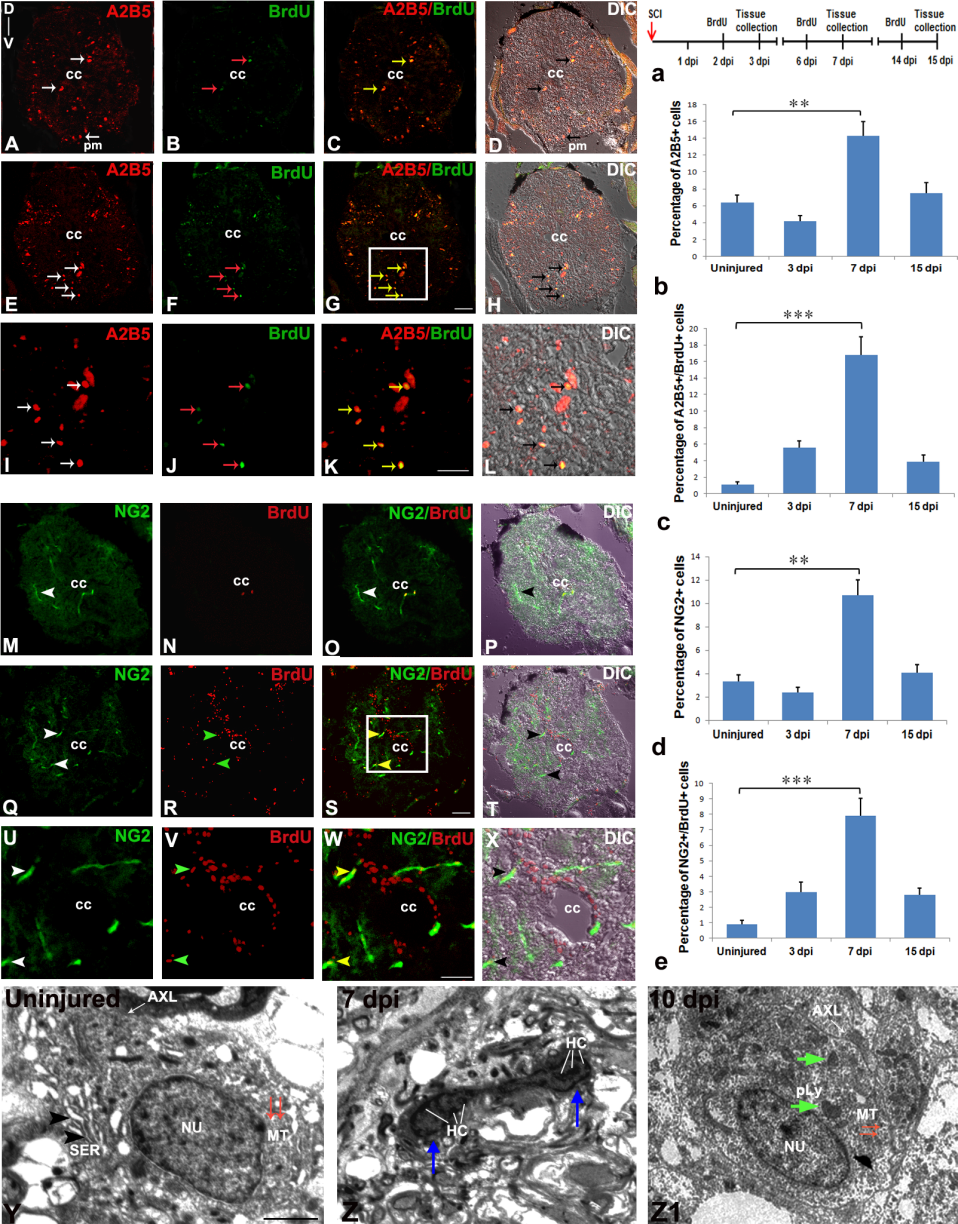
Immunohistochemical and ultrastructural analysis of glial progenitors in uninjured and injured zebrafish spinal cord **:** A-D) A cross section of uninjured cord showing A2B5^+^ cells (white arrows) in white matter and pial membrane (pm, A and D); BrdU^+^ cells (red arrow, B); A2B5^+^/BrdU^+^ colocalized cells (yellow arrows, C) and DIC image of section C (D). E-H) A 7 dpi cord section showing many A2B5 positive cells (white arrow) in white matter and very few in grey matter (white arrow, E); BrdU^+^ cells (red arrow, F); A2B5^+^/BrdU^+^ colocalized cells (yellow arrow, G) and DIC of section G (H). I-L) Higher magnification of boxed area in G showing A2B5^+^ cells (white arrows, I); BrdU^+^ cells (red arrows, J); A2B5^+^/BrdU^+^ colocalized cells (yellow arrows, K) and DIC image of section K (L). M-P) An uninjured cord section showing NG2^+^ bipolar cells (white arrowhead) in the white matter of the cord (M); BrdU^+^ positive cells (N); NG2^+^/BrdU^+^ colocalized cells (yellow arrowhead, O) and DIC image of section O (P). Q-T) A 7 dpi cord section showing many NG2^+^ cells (white arrowheads) in white matter (Q); BrdU^+^ cells (green arrowhead, R); NG2^+^/BrdU^+^ colocalized cells (yellow arrowhead, S) and DIC image of section R (T). U-X) Higher magnification of boxed area in S showing NG2^+^ cells (white arrowheads, U); BrdU^+^ cells (green arrowheads, V); NG2^+^/BrdU^+^ colocalized cells (yellow arrowheads, W) and DIC image of section W (X). Y) Ultrastructure of an uninjured cord section showing an oligodendrocyte with many cisternae (black arrowheads) of smooth endoplasmic reticulum (SER), microtubules (MT, red arrows) in the cytoplasm but no intermediate filaments. Z) A 7 dpi cord section showing a proliferating astrocyte progenitor with a prominent dumb-bell shaped compact dividing nucleus (blue arrows) with characteristic heterochromatin body (HC) in the injury site. Z1) A newly formed oligodendrocyte with small cytoplasmic area where microtubules (MT, red arrows) are obvious and a few cytoplasmic organelles (green arrows) like primary lysosome (pLY) is present at the injury site of 10 dpi cord section. Note that axolemma (AXL) is in close vicinity of the cytoplasm. ‘NU’ indicates nucleus of the cell. a) Drawing represents the time frame of SCI, BrdU treatment and tissue collection for cell proliferation experiments. b-e) Quantification of A2B5^+^ cells (b), A2B5^+^/BrdU^+^ cells (c), NG2^+^ cells (d), NG2^+^/BrdU^+^ cells (e) in uninjured, 3 dpi, 7 dpi and 15 dpi cord. The values in bar represents Mean ± s.e.m. (n = 5), statistical significance represented as p value (Student’s t-test; **p<0.01, ***p<0.001). ‘cc’ denotes central canal of the cord. Scale bar = 50 μm (A-H, M-T); 20 μm (I-L, U-X); 1 μm (Y, Z, Z1).

NG2, a sulfated proteoglycan, is a marker of oligodendrocyte progenitor cells (OPC) [58,59] during CNS development. To identify the nature of proliferating glial progenitors in CNS injury, we have used both A2B5 and NG2 as markers along with BrdU incorporation (Fig 2, S2 Fig and S3 Fig). A2B5 is expressed in both uninjured and injured cord of a zebrafish, indicating the presence of restricted glial progenitor even in an adult cord. The A2B5 expressions are mostly located in white matter but very few cells are also in grey matter. Interestingly, few cells near the pial membrane are also expressing A2B5 (Fig 2A and 2D). The number of A2B5 immunoreactive cells decrease immediately after injury in 3 dpi cord, followed by a significant increase (approximately 8%) in 7 dpi cord compared to uninjured cord (Fig 2B, S2 Table). In 10 and 15 dpi cords, the number of A2B5 immunoreactive cells gradually decreases. When we compare A2B5 immunoreactivity after giving BrdU incorporation, the percentage of dividing A2B5^+^ cells (A2B5^+^/BrdU^+^) are very low both in uninjured (~1%) and 3 dpi cord (~5%), but a significantly higher numbers (~16%, p<0.001) are present in 7 dpi cord (Fig 2C). Similarly, we found very few A2B5 immunoreactive cells are colocalized with GFAP in uninjured cord, and many of the same population also exist primarily in the white matter of 7 dpi cord (S2A, S2B, S2C and S2D Fig). The NG2 immunoreactivity is found in elongated cells, many of which display characteristic bipolar morphology in both adult uninjured and 7 dpi cord (Fig 2M and 2U, S2E and S2E1 Fig). Although NG2 is expressed in the endothelial cells of blood vessels, but these cell are not counted and are excluded from this study. Approximately 3.3% of cells in uninjured cord are NG2 immunoreactive and do not colocalize with GFAP, a known glial marker (Fig 2M, S2 Table). Similar to A2B5, expression of NG2 is also downregulated in 3 dpi cord (Fig 2D) and significantly upregulated (~10%, p<0.01) in 7 dpi cord (Fig 2Q) when compared to uninjured cord (Fig 2D, S2 Table) and are mostly distributed in white matter (Fig 2Q). Percentage of proliferating NG2^+^ cells (NG2^+^/BrdU^+^) cells in uninjured is minimal (~0.9%), whereas in 3 dpi cord nearly 3% cells are expressing both the markers. A relatively high number of proliferating NG2^+^ cells (~8%) are present in 7 dpi cord (Fig 2D, S2 Table). Very few cells express both NG2 and A2B5 are also present in -injured cord (Fig 2E, 2F and 2F1, S2E and S2F Fig). Furthermore, TEM analysis revealed that there is presence of proliferating astrocyte progenitors in 7 dpi cord, with typical heterochromatinised body in the nucleus and electron lucent cytoplasm containing very few organelles (Fig 2Z; [60]). These data further supports our immunohistochemical analysis. We also observed the presence of mature oligodendrocyte in uninjured cord with smooth endoplasmic reticulum, microtubules and axolemma in close vicinity of cytoplasm (Fig 2Y) and newly formed oligodendrocyte(s) in regenerating cord (Fig 1Z) as revealed by their appropriate characters like electron lucent cytoplasm, few organelles like smooth endoplasmic reticulum, primary lysosome and presence of microtubules but absence of intermediate filaments unlike astrocytes [61].

### Presence of Schwann cells in injured spinal cord

**Fig 3:**
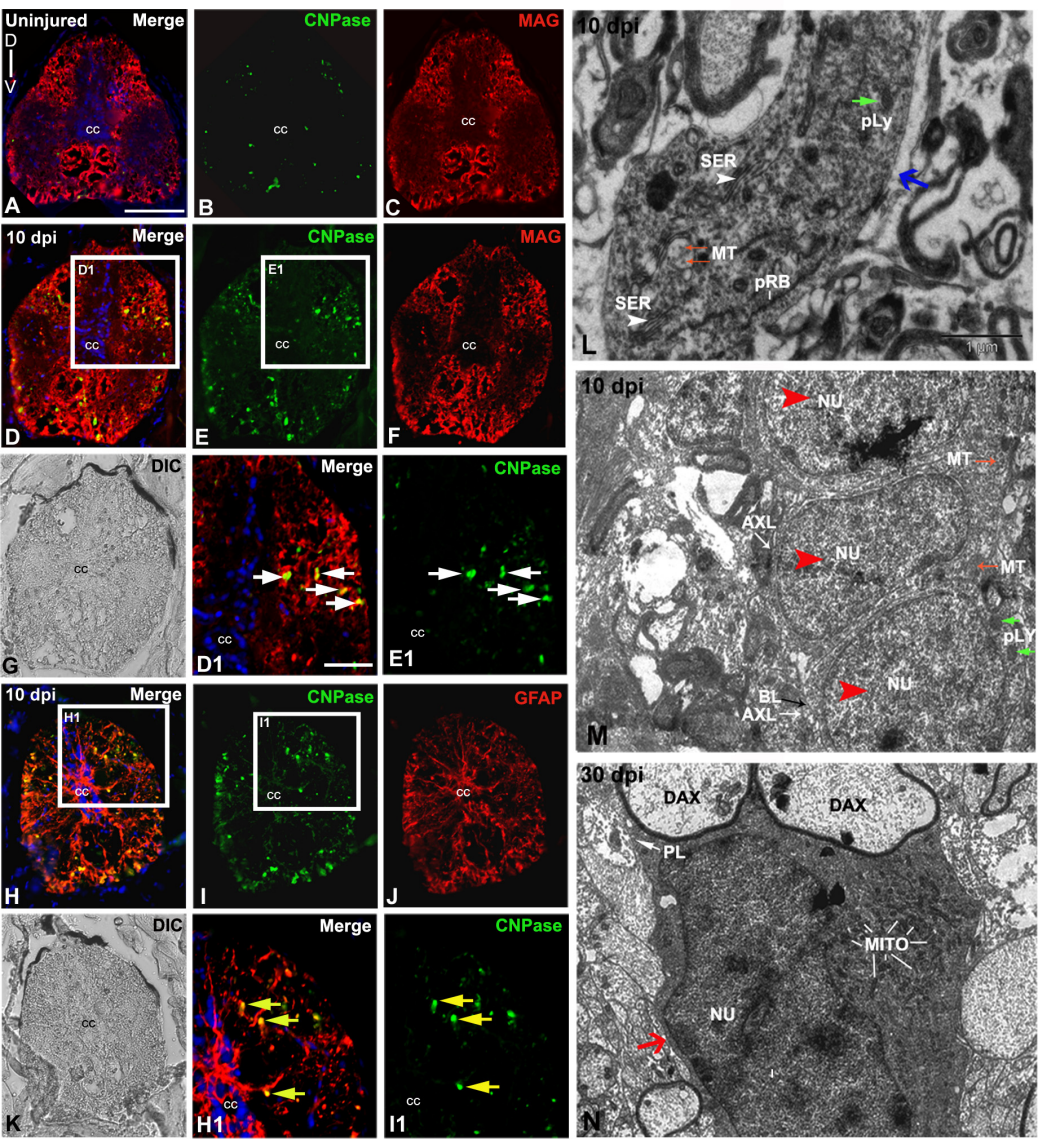
Analysis of myelinating cells in uninjured and injured zebrafish spinal cord **:** A-C) A transverse section of uninjured cord stained with CNPase/MAG/DAPI (A), only CNPase (B) and only MAG (C) respectively. D-G) A 10 dpi cord section stained with CNPase/MAG/DAPI (D) and only CNPase and MAG respectively (E-F) and its DIC image (G) D1) Higher magnification of boxed area in section D showing many colocalized CNPase^+^ and MAG^+^ cells (white arrows) in the white matter. E1) Higher magnification of boxed area in section E, showing CNPase^+^ cells (white arrows) are in the white matter. H-K) A 10 dpi cord section stained with CNPase/GFAP/DAPI (H) and only CNPase and GFAP respectively (I-J), same DIC image (K). H1) Higher magnification of boxed area in section H showing colocalized CNPase^+^ and GFAP^+^ cells (yellow arrows). I1) Higher magnification of boxed area in section I showing CNPase^+^ cells in the white matter. L) Ultrastructure of an oligodendrocyte in the injury site of a 10 dpi cord (blue arrow) with presence of characteristic cisternae of smooth endoplasmic reticulum (white arrowheads, SER), microtubules (MT, red arrows), primary lysosome (pLY, green arrow) and polyribosomes (pRB). Note that the cytoplasm of this oligodendrocyte is without demyelinated axon fibers hence may not actively involved in remyelination process. M) Ultrastructure of a 10 dpi cord section shows three newly formed Schwann cells (red arrowheads) with small cytoplasmic area and nucleus (NU), high N/C index, very thin intercellular spaces and basal lamina (BL, black arrow) close to axolemma (AXL, white arrow). Some cells also have microtubules (MT, red arrows) and few cytoplasmic organelles like primary lysosomes (pLY, green arrows). N) A 30 dpi cord section shows a Schwann cell (red arrow) with a prominent nucleus (NU) remyelinating two demyelinated axons (DAX) surrounded by plasmalemma (PL). The cytoplasm also contains many mitochondria (MITO). ‘cc’ denotes central canal of the cord. Scale bar = 50 μm (A-K); 20 μm (D1, E1, H1, I1); 1 μm (L, M, N).

We observe the presence of a small population of NG2 immunoreactive cells, which are probably oligodendrocyte progenitors. Oligodendrocytes are known to have been involved in myelination of CNS axons. Our TEM analysis previously demonstrated presence of oligodendrocyte in uninjured cord [6]. Furthermore, there are many newly formed Schwann cells in 10 dpi cord adjacent to regenerating axons indicates involvement of Schwann cells in remyelination process as characterized by TEM in injured cords. To identify both myelinating and non-myelinating cells in injured cord, we have used several markers (Fig 3, S4A Fig), such as CNPase/MAG for myelinating cells and CNPase/GFAP for non-myelinating cells [62,63]. MAG and CNPase expressing cells shown restricted localization in the white matter of the cord and very few CNPase expressing cells are present in the uninjured cord (Fig 3A–3C). For obvious reason myelinating cell populations are greater in injured cord. This is quite evident as we observed significant upregulation (p<0.01) of both CNPase and MAG expression compared to that of uninjured cord (Fig 3D and 3E, S4A Fig). Electron microscopic observations revealed that there is presence of oligodendrocytes in the uninjured cord, but to our surprise in injured cord we see fewer oligodendrocytes without myelinated axon fibers (Fig 3L) but many newly formed Schwann cells (Fig 3M). The basal lamina of these Schwann cells can be seen around the plasma membrane of unmyelinated axon fibers (Fig 3M) in injured cord. These Schwann cells are also shown with myelinated axon fibers, rough endoplasmic reticulum, microtubules, and small mitochondria [64] and are probably the Schwann cell progenitors involved in the remyelination process of denuded axons in regenerating cord (Fig 3N).

### Characterization of radial glia subtypes in uninjured and injured spinal cord

**Fig 4:**
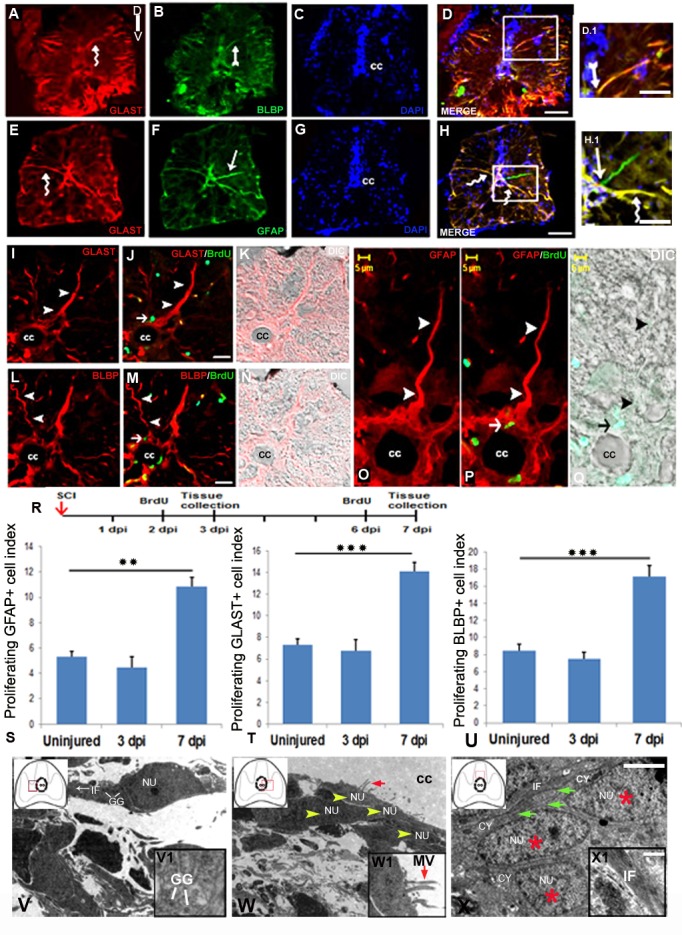
Analysis of radial glial subtypes in uninjured and injured zebrafish spinal cord **:** A-D) Transverse section of an uninjured cord showing GLAST^+^ (A, curved arrow), BLBP^+^ (B, arrow) radial glia counter stained with DAPI (C) and the merge picture (D). D.1) Higher magnification of boxed area of D showing radial glia colocalized with GLAST and BLBP. E-H) Transverse section of an uninjured cord showing GLAST^+^ (E, curved arrow), GFAP^+^ (F, arrow) radial glia stained with DAPI (G) and the merge image (H). H.1) Higher magnification of boxed area in section H showing a radial glia colocalized with GLAST and GFAP (curved arrow), and another radial glia stained with only GFAP (arrow). I-K) A section of 7 dpi cord showing GLAST^+^ radial glia (I, arrowheads), same GLAST^+^ radial glia colocalized with BrdU (J, small arrow) and its DIC image (K). L-N) A 7 dpi cord section showing BLBP^+^ radial glia (L, arrowheads), same BLBP^+^ radial glia colocalized with BrdU (M, small arrow) and its DIC image (N). O-Q) Higher magnification of a 7 dpi cord section showing GFAP^+^ radial glia with its process emerged from central canal (cc) towards pial membrane (O, arrowheads), same GFAP^+^ radial glia colocalized with BrdU (P, arrow) and its DIC image (Q). R) Drawing represents the time frame of SCI, BrdU treatment and tissue collection for cell proliferation experiments. S-U) Representative bar graph indicate quantification of GFAP^+^/BrdU^+^ cells (S), GLAST^+^/BrdU^+^ cells (T), BLBP^+^/BrdU^+^ cells (U) in uninjured, 3 dpi and 7 dpi cord. Values represent as mean ± s.e.m. (n = 5). Statistical significance represented as p value (Student’s t-test; **p<0.01, ***p<0.001). V and V1) Ultrastructure of a young glial progenitor cell with cytoplasmic processes containing intermediate filaments (white arrow, IF) and glycogen granules (white lines, GG) resembling radial glia in the ependymal region of a 7 dpi cord section. Inset V1 showing glycogen granules in the cytoplasm of a radial glia like cells also shown in V. W and W1) Ultrastructure of a 7 dpi cord section at the stump region shows aggregation of many ependymal cells (yellow arrowheads) with conspicuous microvilli (red arrow) protruding towards the central canal (cc). Inset W1 showing higher magnification of an ependymal cells with microvilli (MV, red arrow). X and X1) A 7 dpi cord section in the grey matter region showing many cytoplasmic processes of glia (green arrows) with compact intermediate filaments (IF) in the close vicinity of newly formed cells (red stars). Inset X1 indicating intermediate filaments in glial cytoplasm. In the insets, red boxed area inside the cartoon of injured spinal cord indicates the anatomical location of ultrastructural images. ‘NU’ and ‘CY’ denote nucleus and cytoplasm of the cell anatomy respectively. Scale bar = 50 μm (A-D, E-H); 20 μm (D.1, H.1); 15 μm (I-K, L-N); 5 μm (O-Q); 2 μm (V-X); 200 nm (V1-X1).

Analysis of cortical radial glial cells during development showed lineage heterogeneity, with subtypes of neurogenic and gliogenic radial glia [41]. Proliferating ventricular radial glia serves as precursor and can yield neurons after spinal cord injury [6,13,65,66]. To investigate the presence as well as the role of different subtypes of radial glia in injured cord, we have used different subtype markers of radial glia. Interestingly, all such markers like GLAST, BLBP and GFAP (Fig 4A, 4E, 4B and 4F) are expressed in uninjured spinal cord and the expression is confined in cells with characteristic radial glial morphology (Fig 4I, 4L and 4O). There are three different subtypes of radial glial population namely GLAST^+^/BLBP^+^, GLAST^+^/GFAP^+^ and GFAP^+^/GLAST^-^ exists in uninjured cord (Fig 4D, 4D.1, 4H and 4H.1). The presence of radial glia as proliferating precursor was shown by using BrdU along with other radial glial markers in injured cord. The 7 dpi cord showed BrdU^+^ radial glial cells in the ependyma expressed different markers like GLAST, BLBP and GFAP (Fig 4I–4K, 4L–4N and 4O–4Q respectively).

All these three subtypes of proliferating radial glia like GLAST^+^/BrdU^+^, BLBP^+^/BrdU^+^, GFAP^+^/BrdU^+^ cells are present in uninjured cord and their numbers do not change significantly in 3 dpi cord compared to uninjured cord. Interestingly, their numbers in 7 dpi cord are increased significantly (p<0.01 and p<0.001) and highest for all 3 phenotypes corresponding with highest proliferation rate (Fig 4S–4U). Ultrastructural analysis also confirmed presence of radial glia and many multi-ciliated ependymal cells around the central canal in the 7 dpi cord (Fig 4V and 4W). These radial glial cells with cytoplasmic processes contain intermediate filaments and glycogen granules and the ependymal cells have characteristic multiple cilia ending towards the central canal [67, 68]. These cells also resemble to glial progenitors and may be implicated in migration of newly formed cells adjacent to glial process as shown in Fig 4X.

### Expression of pluripotent stem cell like markers by proliferating cells after SCI

**Fig 5:**
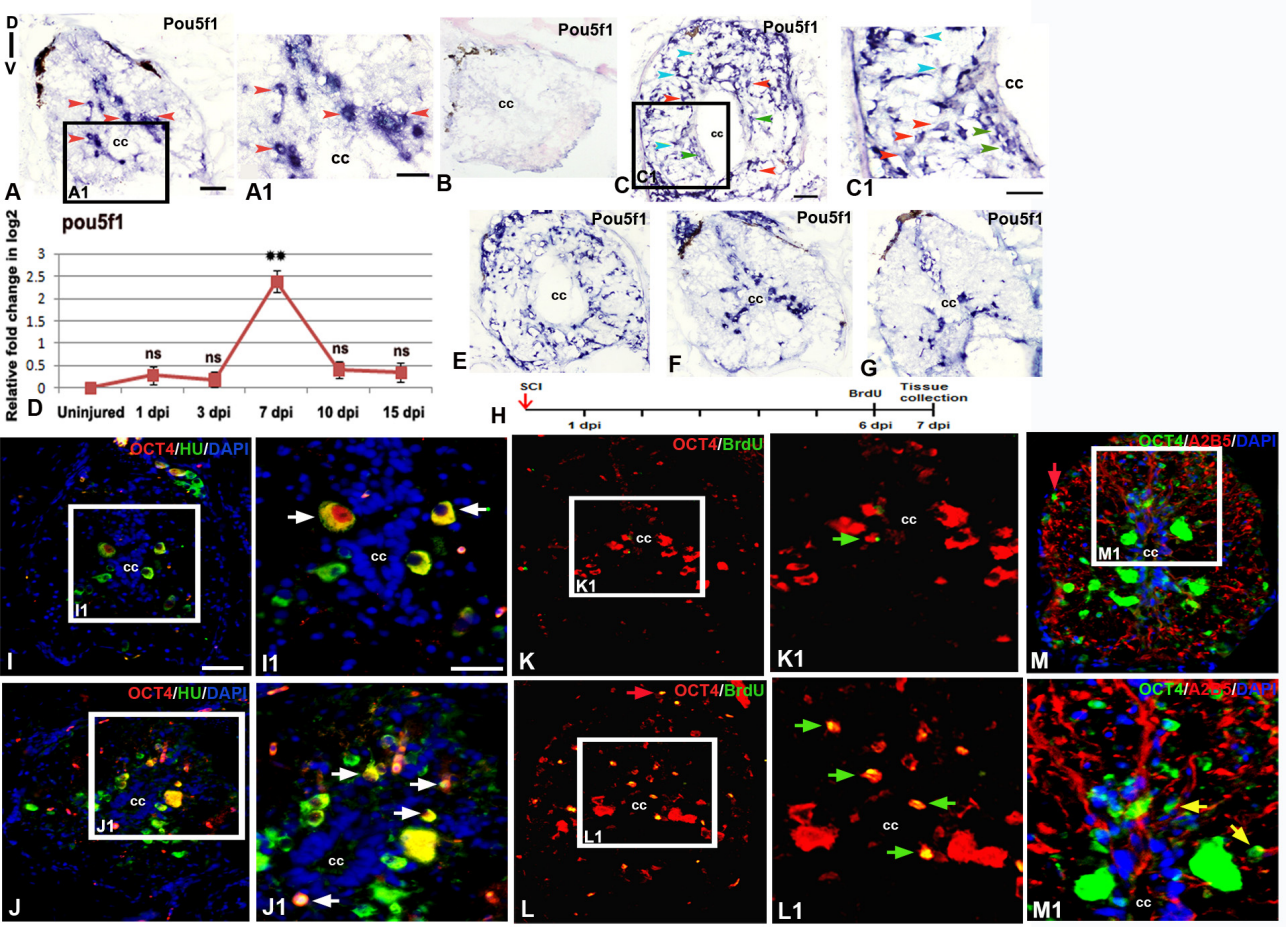
Expression of pluripotency marker in adult uninjured and injured spinal cord **:** A) Localization of *pou5f1* transcripts in adult uninjured cord using a DIG-labeled anti-sense probe. A1) Boxed area of section A at higher magnification shows *pou5f1* expression in many sub-ependymal neurons (red arrowheads). B) Lack of hybridization of a sense probe for *pou5f1* in a 7 dpi cord C) A 7 dpi cord section showing presence of *pou5f1* transcripts in diverse cell types like ependymal cells (green arrowheads) around the central canal (cc), Sub-ependymal neuronal cells (red arrowheads) and glial cells (blue arrowheads). C1) Same cord section at higher magnification. D) Quantitative RT-PCR of *pou5f1* showing fold change (red graph) and pattern of expression at different time points after injury. Error bar indicates the value of s.e.m. (n = 3). Statistical significance of the data represented as p value (ANOVA; **P≤ 0.01, ns = not significant). E-G) Spatial differences in expression pattern of *pou5f* transcripts in a 7 dpi cord near injury epicenter (E), adjacent to injury epicenter (F) and away from injury epicenter (G). H) Drawing represents the time frame of SCI, BrdU treatment and tissue collection for cell proliferation experiments. I &J) Sections show OCT4 protein (encoded by the *pou5f1* gene) expression along with HuC/D immunostaining in uninjured and 7 dpi cord respectively. I1 &J1) Higher magnifications of boxed area in I and J showing OCT4^+^ and HuC/D^+^ colocalized cells (white arrows). K &L) Sections show OCT4 protein expression and BrdU immunostaining in uninjured and 7 dpi cord respectively. K1 & L1) Higher magnifications of boxed area in K and L showing OCT4^+^ and BrdU^+^colocalized cells and are newly born neuron (green arrows). M) A 7 dpi cord section shows OCT4 protein expression and A2B5 immunostaining. M1) Higher magnification of boxed area in L, showing few OCT4^+^ and A2B5^+^ cells (yellow arrows). Note that in section L and M, a few OCT4^+^ cells are also present in pial membrane (red arrows) of the injured cord. “cc” denotes central canal of the cord in all figures. Scale bar = 50 μm (A, B, C, E, F, G, I-M); 30 μm (A1, C1); 20 μm (I1, J1, K1, L1, M1).

Based on our cDNA array analysis [18], we have identified 35 pluripotency related genes expressed in regenerating zebrafish spinal cord. Among these several pluripotency related genes which are also expressed in regenerating fin, limb and tail in different species, we observe upregulation of two common genes *pou5f1* and *sox2*, in regenerating cord (S3 Table). Expression of *pou5f1* which is a zebrafish homolog of mammalian OCT4 [69] has been documented at different time points after injury (Fig 5). Quantitative analysis by qRT-PCR corroborates well with in situ hybridization and immunohistochemistry observations (Fig 5).

The in situ hybridization analysis showed *pou5f1* mRNA is abundant in neuron like cells localized in the subependymal zone in grey matter of uninjured cord (Fig 5A and 5A1). Interestingly, cellular pattern of *pou5f1* expression in 7 dpi cord differs from uninjured one, as it is upregulated in different cell types after injury. High levels of *pou5f1* transcripts are present in the injury epicenter, in 7 dpi cord. *Pou5f1* mRNA expression is also found in the ependymal cells around the regenerating central canal, in newly formed neuron like cells and in other population of glial cells in white matter (Fig 5C and 5C1). In injured samples, a unique observation is the upregulation of *pou5f1* expression in white matter which is more prominent near the epicenter and down-graded at the adjacent region. Expression at far adjacent region is near normal and seen only in grey matter as in uninjured cord (Fig 5F and 5G). The qRT-PCR analysis showed 2.8 fold increase of *pou5f1*mRNA expression in 7 dpi cord than uninjured cord (Fig 5D). However, *pou5f1* transcripts are reduced in 3 dpi cord and 10 dpi cord (Fig 5D). The level of expression in 15 dpi is more or less similar to that of uninjured cord (Fig 5D). We have later characterized protein level expression of OCT4 by using antibody along with HuC/D, a neuronal marker and a proliferation marker, BrdU (Fig 5, S6 Fig and S4B Fig). We found that there are OCT4^+^/HuC/D^+^ neurons in the grey matter of uninjured cord (Fig 5I and 5I1). The number of OCT4 expressing cells increased significantly in 7 dpi cord, both in grey and white matter (Fig 5J and 5J1) and this data also corroborates well with the in situ hybridization analysis of *pou5f1*. In uninjured cord, the neurons are OCT4^+^/BrdU^-^, although a very few colocalized cells are in grey matter (Fig 5K and 5K1). A sharp rise in the number of OCT4^+^/BrdU^+^ cells in 7 dpi cord suggests OCT4 is associated with proliferating progenitors (Fig 5L and 5L1). In 7 dpi cord, many OCT4^+^/HuC/D^+^ colocalized cells are present in grey matter (Fig 5J1), and are similar to small newborn neurons as confirmed by observing the morphology and ultrastructure in our earlier analysis [6]. There are cells near subpial membrane which are also OCT4^+^/BrdU^+^ and a few OCT4 immunoreactive cells present in white matter, co-express A2B5 (Fig 5L and 5M).

### Expression of Vimentin and Msx in neural progenitor

Our cDNA array data [18] refers to expression of several genes related to dedifferentiation mechanism and epithelial-mesenchymal (E-M) transition event. We have studied expression of vimentin [44] and Msx-1 [70–72] as markers for dedifferentiation and E-M transition [46,73].

**Fig 6:**
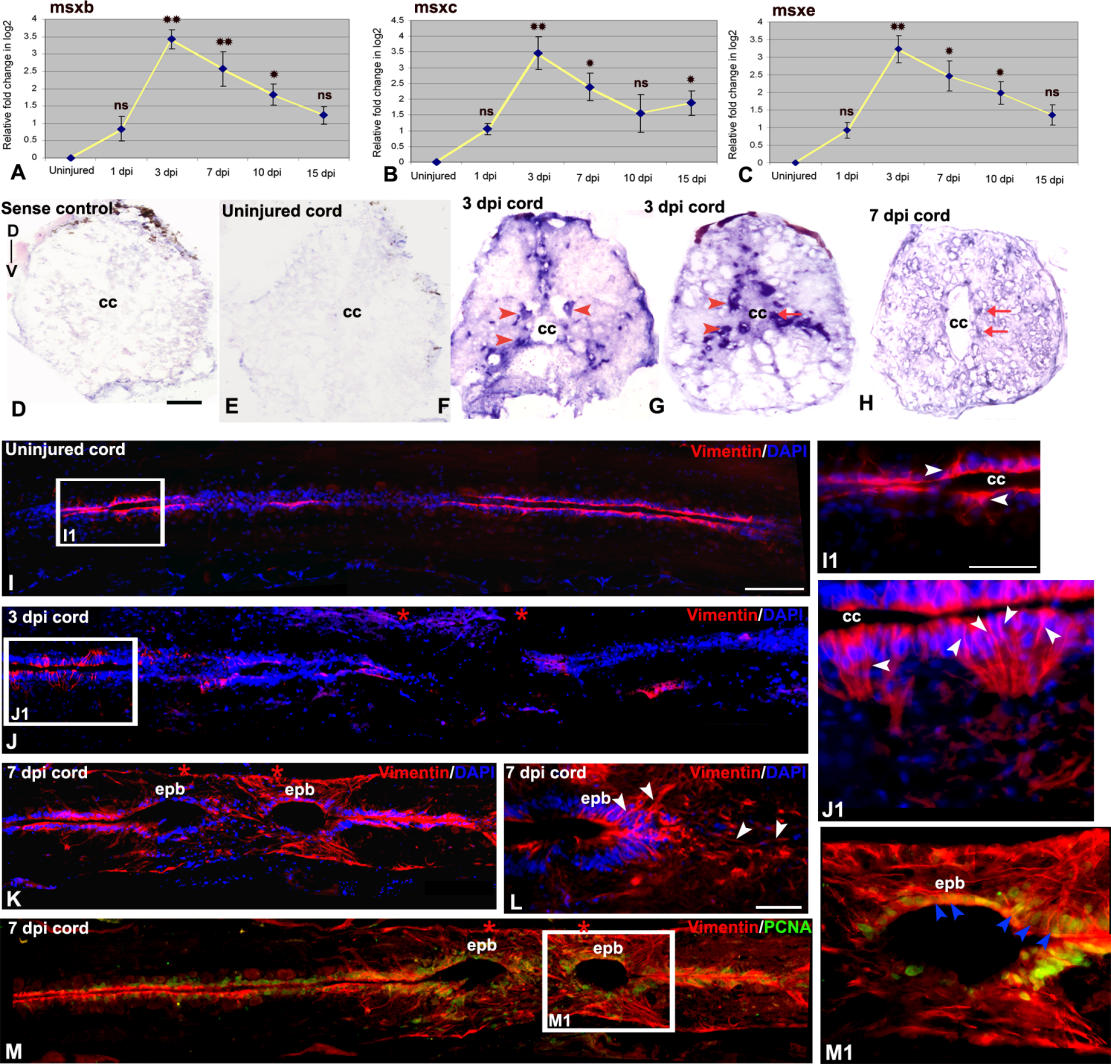
Expression of Vimentine and Msx in adult uninjured and injured spinal cord **:** A-C) Quantitative RT-PCR of *msx-b*, *msx-c and msx-e* expression showing fold change (yellow line graph) and temporal expression pattern after injury. Error bar indicates the value of s.e.m. (n = 3) and statistical significance represented as p value (ANOVA; **P≤ 0.01, *P≤0.05, ns = not significant). D) Lack of hybridization of a sense probe for *msx-b* in a 3 dpi cord. E) A transverse section of uninjured cord shows no expression of *msx-b* transcripts. F&G) Two representative transverse sections of 3 dpi cord shows presence of *msx-b* transcripts using a DIG-labeled anti-sense probe. *msx-b* transcripts are localized both in subependymal region, which houses mostly neurons (Hui et al., 2010) (red arrowheads) and in ependymal region around the central canal (‘cc’; red arrow). H) A 7 dpi cord section showing presence of *msx-b* transcripts in the ependymal cells around the central canal (‘cc’; red arrow). I) A longitudinal section of uninjured cord stained with vimentin and DAPI. I1) Higher magnification of boxed area in section I, shows vimentin positive cells (white arrowheads) present exclusively in ependyma around the central canal (cc). J) A 3 dpi cord section stained with vimentin and DAPI. Note that vimentin expression is lost in the injury epicenter (double red star) but present in normal part of the cord. J1) Higher magnification of boxed area in section J, shows many vimentin positive cells (white arrowheads) around the central canal (cc). K) A longitudinal section of 7 dpi cord showing upregulation of vimentin expression in the ependymal bulb (epb) in the injury epicenter (double red star). L) Another 7 dpi cord section showing vimentin expression (white arrowheads) in ependymal bulb (epb) and surrounding mesenchyme. M) A 7 dpi cord section stained with vimentin and PCNA. M1) Higher magnification of boxed area in section M, shows many vimentin^+^/PCNA^+^ cells (blue arrowheads) in the ependymal bulb (epb). Scale bar = 20 μm (I1, J1, M1); 50 μm (D-G, L); 100 μm (I-K, M).

Expression of Msx-1 was evaluated by different methodologies such as qRT-PCR, in situ hybridization (Fig 6) and ELISA (S4C Fig). All the three zebrafish homologs of *msx* genes (*msx-b*, *msx-c and msx-e*) showed similar expression pattern in injured state (Fig 6A–6C). A high level of *msx-b* expression was observed in the 3 dpi cord but decreased in 7 dpi cord (Fig 6A) as revealed by qRT-PCR. Similarly, in situ hybridization analysis showed no expression of *msx-b* transcript in uninjured cord (Fig 6E), upregulation in 3 dpi cord, predominantly in grey matter cells and it was decreased in 7 dpi cord (Fig 6F–6H).

Immunohistochemical localization shows presence of vimentin in the cells in ependymal layer around the central canal of uninjured cord (Fig 6I and 6I1), whereas in 7 dpi cord upregulation of vimentin expression is observed in the cells in ependymal layer forming the ependymal bulb–a structure widely recognized in injured cord in both urodele [43] and fish [6] (Fig 6K and 6L). There is an initial loss of expression of vimentin in 3 dpi cord in the injury epicenter (Fig 6J), followed by an increased filamentous nature of expression in 7 dpi cord compared to uninjured cord (Fig 6K–6M). Expression of vimentin is associated with radial glia like cells around the central canal and with the mesenchymal cells around the ependymal bulb (Fig 6L). Many of these vimentin positive cells are in proliferating state and hence colocalized with PCNA in the 7 dpi cord (Fig 6M and 6M1).

## Discussions

### Presence of proliferating neural progenitors

CNS regeneration in zebrafish involves both axonal regrowth and reorganization of ependymal cells that proliferate, migrate and differentiate to give rise to the lost tissue. One of the primary responses following an injury is the proliferation that would eventually yield to both neuronal and glial cells. Adult CNS harbors a slowly dividing quiescent cell population but upon injury, these cell enters cell cycle, hence become transit amplifying and hence rapidly dividing [6,7,36]. Here we provide information on characters of different proliferating progenitors involved during neurogenesis and gliogenesis in the spinal cord of zebrafish before and after a crush injury.

### Proliferating progenitors are Sox2 positive

Injury induced proliferation in fish cord occur both in grey as well as in the white matter of the cord and proliferation can be detected in 3 dpi cord, but the proliferative response peaks at 7 dpi cord [6]. Here we found that after injury almost all cells present around the ependyma are expressing Sox2 and some are expressing proliferation markers. Similarly, Sox2 expressing progenitor cell population also exists in fish optic tectum [14]. In the brain, these Sox2^+^/PCNA^+^ population of cells are capable of self-renewal and hence referred as NSC [35]. Attempt has been made, to identify and analyse NSC in fish brain to exploit their potential to become different types of neural cells [5,14,36]. Sox2^+^ cells are also thought to be neural progenitors in amphibian cord [30,48,74,75]. Radial glia, expressing GFAP, vimentin and aromatase are known to be involved in generation of new neurons [34,44,45,76,77] and providing vital support to migrating neurons [78,79]. Deletion of Sox2 in the axolotl cord resulted in defective proliferation of GFAP^+^ cells. It has been suggested that Sox2 mediate expansion of neural stem cell pools are required for regenerating axolotl spinal cord [74]. Others reported Sox2 and GFAP are expressed in ependymo-radial glia in adult newt brain and they are in proliferating state [80]. Presence of both Sox2^+^/GFAP^+^ ependymo-radial glia and Sox^+^/NeuN^+^ cells in subependyma have been demonstrated in axolotl spinal cord after transection injury [30]. We reported earlier the presence of Sox2^+^/BrdU^+^ cells in regenerating spinal cord, where we studied the expression of Sox2 to validate our cDNA array hybridization data [18]. In the present analysis, we have found that a significant rise of Sox2^+^/BrdU^+^ cells (8–12%) in injured cord suggesting the presence of NSC like proliferating progenitor during regeneration of adult zebrafish spinal cord. There is also a high fold increase (~5 fold) in *sox2* mRNA level in injured cord (3 dpi) compared to uninjured cord. Further immunohistological analysis also supported our qRT-PCR analysis. Since we found that 6–9% of Sox2 expressing cells are also HuC/D positive, a marker expressed in newborn neurons, indicating that Sox2 positive cells are contributing to regenerative neurogenesis. [19] also reported upregulation of Sox2 in ependymal cells following transection injury in zebrafish spinal cord and suggested its role as proliferation initiator in ependymal cells as these Sox2^+^ cells also expressed PCNA, another proliferation marker at later time point of regeneration. Although they showed no evidence to suggest that these cells are expressing neuronal marker after injury and thus contributing to regenerative neurogenesis. While experiment involving regeneration competent *Xenopus* larvae demonstrated massive proliferation Sox2/3^+^ cells following injury, suggesting occurrence of neurogenesis as there are upregulations of several markers, such as doublecortin, α-tubulin [75].

The expression of Sox2 in our analysis identifies a particular progenitor and is associated with neurogenic state of the progenitor, which was not shown earlier in regenerating zebrafish spinal cord [19]. These proliferating progenitors indeed generate new neurons as confirmed by the neuronal marker. Identification of newly born neurons and their morphological resemblance also corroborates with our previous analysis of the cellular identity in regenerating cord [6]. Apart from HuC/D^+^ neuronal cells, GFAP^+^ radial glias also express Sox2. Expression Sox2 in radial glia is probably related to neurogenic potential of radial glia [41,81] as shown in regenerating axolotl cord [30,74].

### Presence of different types of glial progenitors

We have identified glial restricted precursors (GRP) of both astrocytic lineage and oligodendrocytic lineage by using A2B5 and NG2 in fish cord. GRP are also found in developing mammalian neural tube and that can give rise to astrocytes and oligodendrocytes [55,56]. A2B5 also represents O-2A progenitors and defines an intermediate glial precursor in embryonic cord [82–85]. We have shown that the number of A2B5 cells decrease after injury probably because there is initial tissue loss immediately after injury. Although injury epicentre of 3 dpi cord shows presence of some cells because of cell migration and proliferation and it continues until 7 dpi cord, where we see plenty of accumulated cells at the injury epicentre. The number of proliferating A2B5 expressing cells increased significantly in 7 dpi zebrafish cord. These cells are mostly present in white matter and may probably generate cells of astrocyte lineage. We found a good percentage of A2B5 immunoreactive cells are also expressing GFAP, as observed by others in developing mammalian CNS [86]. The presence of astrocyte precursors in injured zebrafish cord was further confirmed by TEM, where we have shown the presence of proliferating astrocyte progenitors in 7 dpi cord.

Recently it has been reported that A2B5^+^ cell line generated from another teleost brain exhibited markers for both astroglia and oligodendrocytes [33], although we observed very few A2B5^+^ cells expressing NG2 –a marker for oligodendrocyte progenitor in injured cord. Others [87] reported that in adult human brain A2B5^+^ progenitors could undergo a limited number of cell divisions *in vitro* and can generate oligodendrocyte. Adult A2B5^+^ cells retain properties of progenitor cells when compared to post mitotic mature human oligodendrocytes and are more committed to the oligodendrocyte lineage than their fetal counterparts [88]. In adult teleost fish CNS, there is heterogeneity of stem cell; an overwhelming majority of adult stem cell in brain stem /spinal cord area is glia [89]. However, in regenerating zebrafish cord, only a minor population of A2B5 expressing cells colocalize with NG2. Our data also suggests that both A2B5 and NG2 expressing progenitor like cells exist in adult uninjured cord indicative of its embryonic character in the adult [90,91].

In mammalian SCI, upregulation of NG2 was observed in the oligodendrocyte progenitors and macrophages near injury epicenter [92]. In injured fish cord increase of both proliferating A2B5 and NG2 immunoreactive cells suggest injury induces proliferation of these glial progenitors. Both populations identify GRP like cells which are present predominantly in white matter. The cell type expressing NG2 in regenerating cord are few small cells and many elongated cells with long bipolar processes, unlike developing mammalian CNS, where large cells with multiple processes were observed [86]. It would be important to analyse the fates of various glial restricted precursors in fish spinal cord, since identity of these precursor may vary from mammalian counterpart, where new cells are generated from astrocytic stem cell [4,93,94]. Most importantly these glial precursors can respond to injury and participate in repair process in fish cord. Furthermore, unlike mammal, in teleost cord it has been reported recently that FGF dependent glial bridge formation facilitates axonal regeneration, suggesting that gliosis at injury site augments regeneration in fish cord [34].

### Radial glia represents a heterogeneous population

We showed the presence of radial glia and its injury induced proliferation in adult cord [6], where we showed both slowly dividing and rapidly dividing cells. Among the radial glia in adult CNS, some are slowly dividing, at quiescent state, have capacity for self-renewal and while others can produce transient amplifying neuronal precursors during neurogenesis [95]. Therefore, dividing radial glia is not a homogeneous population. These cells are also associated with the remarkable ability of the CNS regeneration both in urodele and fish CNS [68,96]. Radial glial cells are neurogenic in a number of non-mammalian CNS both in adult and developing stage [79, 97]. In this context it is important to mention that in mammalian CNS, radial glia can come from multipotent neural stem cells and represent the remnant of stem cells in adult CNS and can generate both astrocytes and neurons [98,99]. Adult zebrafish telencephalon harbors heterogeneous population of radial glia with respect to their rates of division and expression of different markers like GFAP, GLAST, BLBP and RC2 [5,9,10,41]. We have identified different subtypes of radial glia in zebrafish cord before and after a crush injury. In uninjured cord these cells are present surrounding the ependymal canal and express markers like GFAP, BLBP and GLAST. Following an injury, increased incorporation of BrdU in cells expressing these markers suggests that many radial glial cells had started to proliferate. These radial glial cells in the zebrafish cord have clear ependymal feature, and possess motile cilia as observed in our TEM analysis and confirmed by others [68].

At least three different types of radial glial populations namely GLAST^+^/BLBP^+^, GLAST^+^/GFAP^+^ and GFAP^+^/GLAST^-^ are present in uninjured cord. We also confirm the heterogeneity among these glial populations even when these are in proliferating state after injury. Radial glia are neurogenic in developing mammalian CNS [95,100] and others have shown that differentiated glia can express high level of GFAP and activated glia loose GFAP expression. These cells can also dedifferentiate to generate neurons and radial glia following injury induced proliferation in zebrafish cord [13,34]. Approximately 8% of the proliferating cells in zebrafish spinal cord differentiate into newly generated motor neurons after transection injury [13]. The ependymo-radial glia or radial glia in adult zebrafish CNS display key features of stem cells, like slowly dividing quiescent population, self-renewal and generation of different cell types [6,13,101]. Furthermore, distinct domain of ependymo-radial glia in adult injured cord can give rise to different types of neurons as different domains are specified by different combination of transcription factors. For examples motor neuron progenitor (pMN) like domain would express olig2/nkx6.1/pax6 and would generate motor neurons [68,102]. All these evidence indicates involvement of radial glia in neurogenesis of regenerating zebrafish cord.

Vimentin, an intermediate filament protein, labels radial glia in developing cortex [79,81]. In regenerating urodele cord, it is considered to be a NSC marker [44] and associated with epithelial-mesenchymal transition [43]. Expression of vimentin by injury-reactive ependymal cells in urodele cord was interpreted as the creation of embryonic environment where we see dedifferentiation and return of multipotent state progenitors [44]. In zebrafish cord, we show similar expression of vimentin in radial glia situated in the ependymal layer. An increased level expression of dividing radial glia with vimentin in injured cord indicates that the most dividing progenitor radial glia express vimentin.

### Expression of markers related to cellular dedifferentiation and reprogramming in regenerating cord

Cellular dedifferentiation is a common phenomenon during the regeneration of various organs [29]. Epimorphic regeneration in urodele amphibians and teleost fish involve dedifferentiation, where terminally differentiated cells can convert to undifferentiated or less differentiated progenitors [103–105]. In zebrafish, dedifferentiation represents a key phenomenon during regeneration of heart, retina and fin where blastemal cells show lineage restriction [106–108]. Increased expression of transcription factor msx-1 is responsible for driving the terminally differentiated state towards undifferentiated state through the process of dedifferentiation [21, 109]. During the process of dedifferentiation, proliferation provides the basis for tissue regeneration and creation of new cell lineages [29]. Msx also keeps the blastemal cells in several organs in proliferating state [110–112]. Interestingly in *Xenopus*, Msx promotes spinal cord regeneration even during refractory period, from stage 45–47 [113]. Both *msx-b* (zebrafish homologue of Msx-1) and vimentin are upregulated in proliferating cells near or in the ependymal layer in zebrafish cord and thus suggesting a possible involvement of ependymal cells in the dedifferentiation process. Another possibility is the involvement of Schwann cell as a dedifferentiating cell population [29,106]. During development, Schwann cell precursor can dedifferentiate and redifferentiate to mature cells [114]. Similar presence of dedifferentiating and proliferating Schwann cells has been observed after nerve injury [115]. However, remyelinating Schwann cells in CNS are known to migrate from peripheral nervous system through spinal and cranial roots, meningeal fibres or autonomic supplies [116]. Presence of Schwann cell progenitors in regenerating zebrafish spinal cord is a unique feature, never been reported earlier and are involved in remyelination process as shown by TEM analysis. Although we cannot ascertain the origin of Schwann cells in injured spinal cord but we show presence of newly formed Schwann cells in the injured cord that may have generated through dedifferentiation and proliferation of mature Schwann cells. In future role of Schwann cell in regenerating cord need s to investigated, since these cells may play important role in creating regeneration permissive niche.

Another promising procedure to be exploited for regenerative therapies is cellular reprogramming where somatic cells can be converted into stem cells. Reprogramming process is known to involve four core transcription factors Oct4 (zebrafish homologue of Pou5f1), Sox2, cMyc and Klf4 [25,27,117,118]. It has also been suggested that dedifferentiation is a common cellular mechanism during induction of pluripotent stem cells and during appendage regeneration in fish and urodele amphibians [29,103,105]. Whether CNS regeneration in zebrafish is due to presence of resident stem cell population or due to reprogramming of mature cells is not clear. However during appendage regeneration, it has been suggested that both mechanisms i.e., dedifferentiation and activation of stem cell population are involved [119,120]. To understand the origin and fate of the new cells generated during CNS regeneration, the developmental potential (multipotent) of the suggested cell types needs to be explored. Expression of several reprogramming genes has been reported during regeneration of lens, fin and limb [25,121]. Among the four most commonly used reprogramming genes, we have shown upregulated expression of *pou5f1* and Sox2 in neural progenitors which are probably involved in specifying neural progenitors during neurogenesis after injury. Similar role in neurogenesis for Sox2 has also been described in mouse [122] and zebrafish brain [11,36].

Our qRT-PCR analysis and cDNA array data indicate that there is increased expression of *pou5f1* in regenerating zebrafish cord [18]. Others reported, expression of *pou5f1* in normal and regenerating fins concluding that *pou5f1* is required for fin regeneration but not crucial for blastema formation [25]. Three genes *pou5f1*, *sox2* and *msx-b* all are required for the regenerative outgrowth [25]. We did not obtain any blastema like structure in regenerating cord because of the nature of injury but interestingly all these three genes are upregulated after injury suggestive of a common underlying genetic mechanism regulating the regenerative processes in this species. *Pou5f1* is normally expressed in subependymal regions housed by neurons in uninjured cords suggestive of the fact that even uninjured zebrafish cord is neurogenic and *pou5f1* is associated with neurogenesis [38,123]. Upon injury, OCT4 is associated with different kind of cell types initially but expression later becomes concentrated in newborn neuronal cells suggesting their role in neurogenesis. It is unlikely that it confers real pluripotency, although direct reprogramming of human neural stem cells by OCT4 has been reported in vitro [28,124].

In future, the involvement of *pou5f1* and other genes in reprogramming of neural cells in zebrafish should be addressed by designing more thorough genetic and functional analysis. In the present context of understanding the mechanism of in vivo regeneration, we can interpret that regenerating spinal cord harbors multiple proliferating progenitors as identified by us using known markers of different progenitors. There are high levels of injury induced proliferation, both glia and neurons are generated from endogenous progenitor cells and different types progenitors are involved during regeneration of spinal cord. The genes related to cellular dedifferentiation are also expressed in various cell types. Further genetic dissection of endogenous regenerative potential of adult spinal cord would allow us to use zebrafish as an ideal model to facilitate the development of improved therapeutic strategy for CNS injury and neurodegenerative diseases.

## Supporting Information

S1 Fig Immunohistochemical analysis of neural progenitors: A) A transverse section of 7 dpi cord showing many SOX2^+^ cells (white arrow)in gray matter and predominantly around the central canal (cc). B) A 7 dpi cord section shows SOX2^+^/BrdU^+^ proliferating cells (white arrowheads). C) A 7 dpi cord section stained with SOX2 and HuC/D. Subependymal region of the cord shows some colocalized SOX2^+^/HuC/D^+^ cells (yellow arrowheads). D) A 7 dpi cord section stained with SOX2 and GFAP. Some of the SOX2^+^ cells around central canal (cc) are also GFAP^+^ (grey arrowheads). Scale bar = 50 μm (A-D).
(TIF)

S2 Fig Immunohistochemical analysis of glial progenitors: A-B) Transverse section of an uninjured cord stained with A2B5 and GFAP (A) and its corresponding DIC image (B). A1) Higher magnification of boxed area in section A showing A2B5 and GFAP colocalized cells (white arrowheads). C-D) Transverse section of a 7 dpi cord stained with A2B5 and GFAP (C) and its corresponding DIC image (D). C1) Higher magnification of boxed area in section C showing many A2B5 and GFAP colocalized cells (white arrowheads) predominantly in white matter. E-F) A 7 dpi cord section stained with A2B5 and NG2 (E) and its corresponding DIC image (F). E1) Higher magnification of boxed area of section E showing few A2B5^+^ and NG2^+^ cells (yellow arrowheads) in the white matter. ‘cc’ denotes central canal of the cord. Scale bar = 50 μm (A -F); 20 μm (A1, C1, E1).
(TIF)

S3 Fig Immunohistochemistry of negative controls: A-D) Transverse section of a 7 dpi cord stained without primary antibody A2B5 for negative control in injured cord of Fig 2E. No primary antibody A2B5 (A), DAPI (B), Merge (C) and DIC (D). E-H) Transverse section of a 7 dpi cord stained without primary antibody NG2 for negative control in injured cord of Fig 2Q. No primary antibody NG2 (E), DAPI (F), Merge (G) and DIC (H). ‘cc’ denotes central canal of the cord. Scale bar = 30 μm.
(TIF)

S4 Fig Quantification of neural and glial progenitors: A) Quantification of CNPase^+^/MAG^+^ and CNPase^+^/GFAP^+^ cells in uninjured and 10 dpi cord. Values represent as mean ± s.e.m. (n = 3). Statistical significance represented as p value (Student’s t-test; **p<0.01, ‘NS’ denotes not significant). B) Quantification of OCT4^+^/HuC/D^+^, OCT4^+^/BrdU^+^ and OCT4^+^/A2B5^+^ colocalized cells in uninjured and 7 dpi cord. Values represent as mean ± s.e.m. (n = 3). Level of significance represented as p value (Student’s t-test; *p<0.05, **p<0.01). C) Quantitative expression of Msx-1 protein at different time points by using ELISA. Values indicate mean ± s.e.m. (n = 3) and statistical significance shown as p value (ANOVA; *p<0.05).
(TIF)

S5 Fig Western blot analysis in zebrafish tissue: Western blot analysis of MAG, CNPase, NG2 and OCT4 protein in zebrafish uninjured and injured (10 dpi and 7 dpi cord respectively) spinal cord tissues. GAPDH represents as internal loading control for uninjured and respective injured spinal cord tissue.
(TIF)

S6 Fig Immunohistochemical analysis of OCT4: A-D) Uninjured cord section of Fig 5I shown in separate images immunostained with OCT4, HuC/D and DAPI. E-H) The 7 dpi cord section of Fig 5J shown in separate images immunostained with OCT4, HuC/D and DAPI. I-K) Uninjured cord section of Fig 5K shown in separate images immunostained with OCT4 and BrdU. L-N) The 7 dpi cord section of Fig 5L shown in separate images immunostained with OCT4 and BrdU. O-R) The 7 dpi cord section of Fig 5M shown in separate images immunostained with OCT4, A2B5 and DAPI. The insets I, II and III in panel R, indicate three different representative OCT4 and A2B5 colocalized cells. Scale bar = 20 μm.
(TIF)

S1 Table Quantification of Sox2^+^ cells colocalized with BrdU, HuC/D and GFAP in uninjured and injured cord at various time points. Values represented as mean ± s.e.m. (n = 5), Statistical significance as p value (Student’s t-test; **p<0.01, ***p<0.001).
(DOC)

S2 Table Quantification of A2B5^+^, NG2^+^, A2B5^+^/BrdU^+^ and NG2^+^/BrdU^+^ cells in uninjured and injured cord sections of various time points. Values represented as mean ± s.e.m. (n = 5), Statistical significance as p value (Student’s t-test; **p<0.01, ***p<0.001).
(DOC)

S3 Table Comparison of pluripotency related genes expressed in various regenerating tissues in different species. (DOC)
