# Supplementary figures and images for: Characterization of Proliferating Neural Progenitors after Spinal Cord Injury in Adult Zebrafish
Source: PLoS One. 2015 Dec 2;10(12):e0143595. doi: 10.1371/journal.pone.0143595 (PMC4667880; doi:10.1371/journal.pone.0143595)

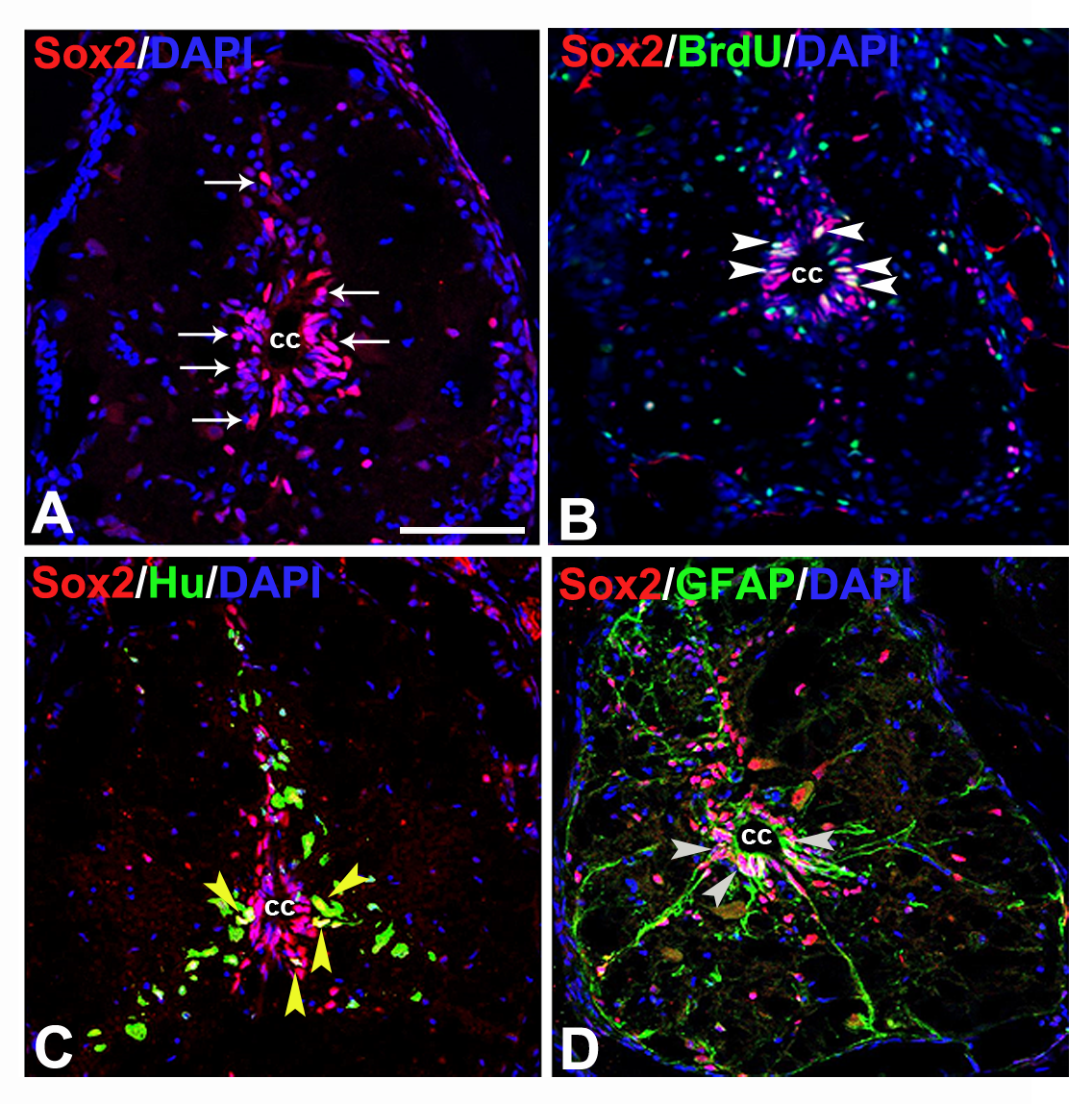

Supplement: S1 Fig — A) A transverse section of 7 dpi cord showing many SOX2+ cells (white arrow)in gray matter and predominantly around the central canal (cc). B) A 7 dpi cord section shows SOX2+/BrdU+ proliferating cells (white arrowheads). C) A 7 dpi cord section stained with SOX2 and HuC/D. Subependymal region of the cord shows some colocalized SOX2+/HuC/D+ cells (yellow arrowheads). D) A 7 dpi cord section stained with SOX2 and GFAP. Some of the SOX2+ cells around central canal (cc) are also GFAP+ (grey arrowheads). Scale bar = 50 μm (A-D). (TIF) [file pone.0143595.s001.tif]

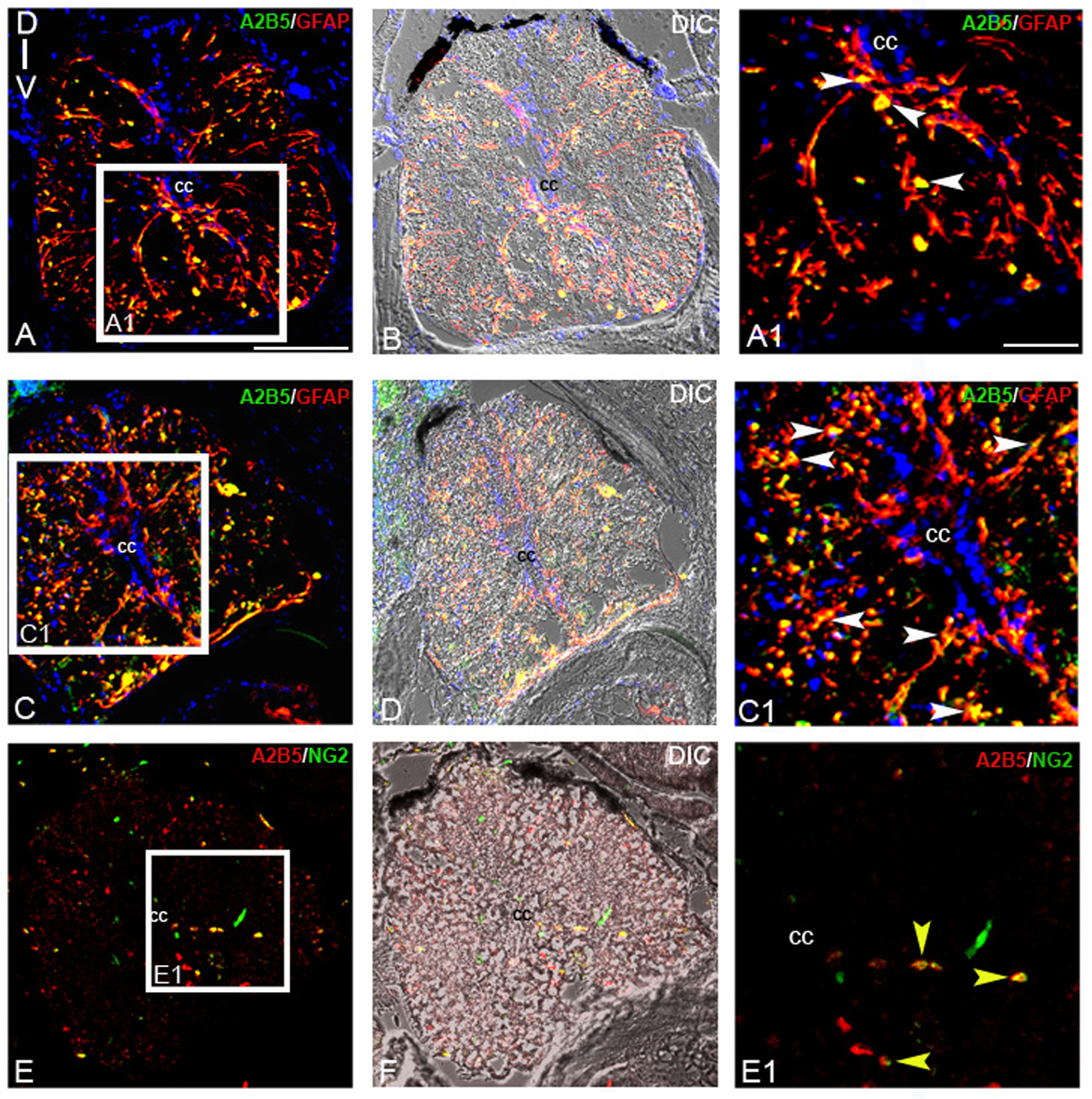

Supplement: S2 Fig — A-B) Transverse section of an uninjured cord stained with A2B5 and GFAP (A) and its corresponding DIC image (B). A1) Higher magnification of boxed area in section A showing A2B5 and GFAP colocalized cells (white arrowheads). C-D) Transverse section of a 7 dpi cord stained with A2B5 and GFAP (C) and its corresponding DIC image (D). C1) Higher magnification of boxed area in section C showing many A2B5 and GFAP colocalized cells (white arrowheads) predominantly in white matter. E-F) A 7 dpi cord section stained with A2B5 and NG2 (E) and its corresponding DIC image (F). E1) Higher magnification of boxed area of section E showing few A2B5+ and NG2+ cells (yellow arrowheads) in the white matter. ‘cc’ denotes central canal of the cord. Scale bar = 50 μm (A -F); 20 μm (A1, C1, E1). (TIF) [file pone.0143595.s002.tif]

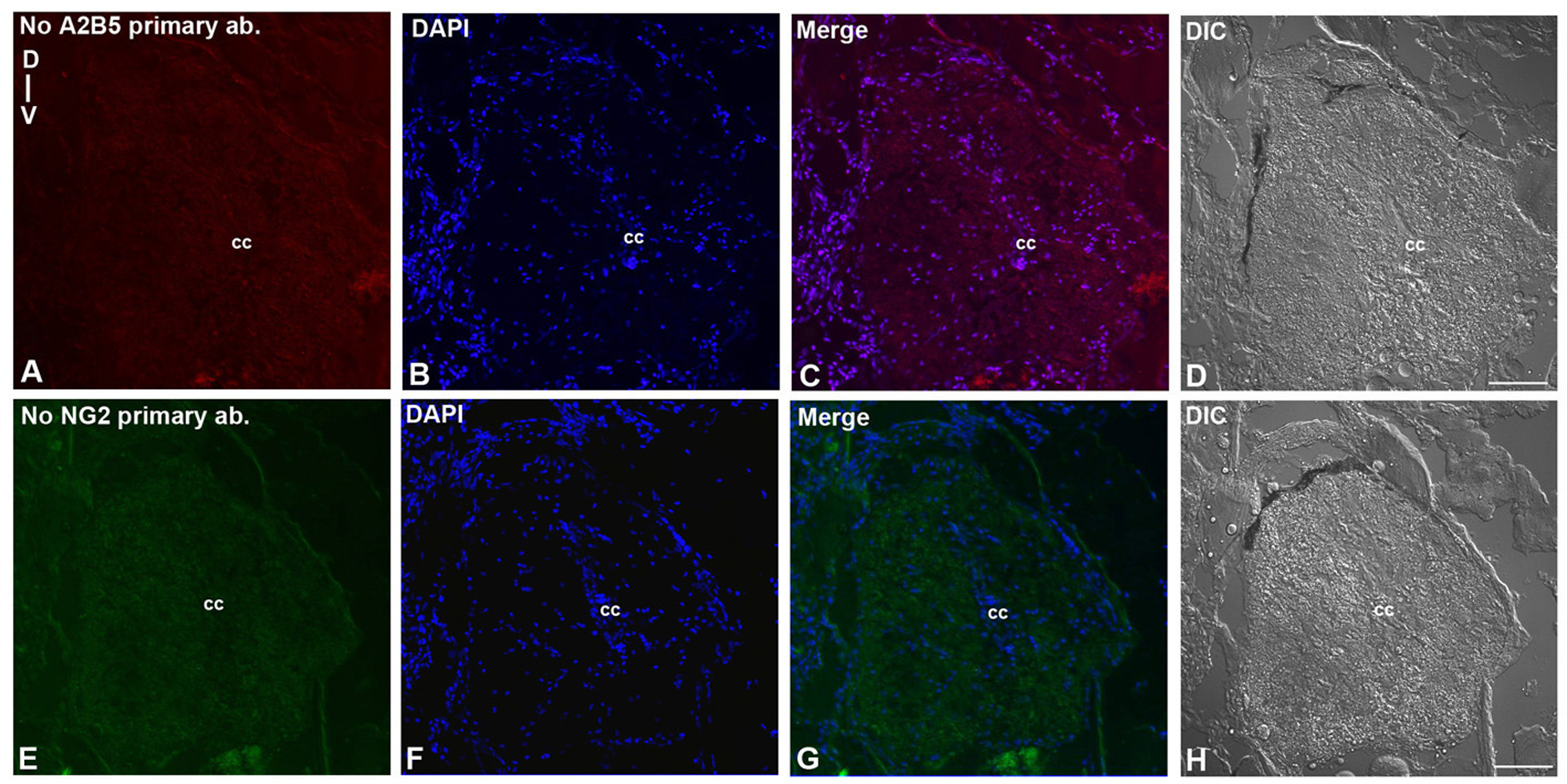

Supplement: S3 Fig — A-D) Transverse section of a 7 dpi cord stained without primary antibody A2B5 for negative control in injured cord of Fig 2E. No primary antibody A2B5 (A), DAPI (B), Merge (C) and DIC (D). E-H) Transverse section of a 7 dpi cord stained without primary antibody NG2 for negative control in injured cord of Fig 2Q. No primary antibody NG2 (E), DAPI (F), Merge (G) and DIC (H). ‘cc’ denotes central canal of the cord. Scale bar = 30 μm. (TIF) [file pone.0143595.s003.tif]

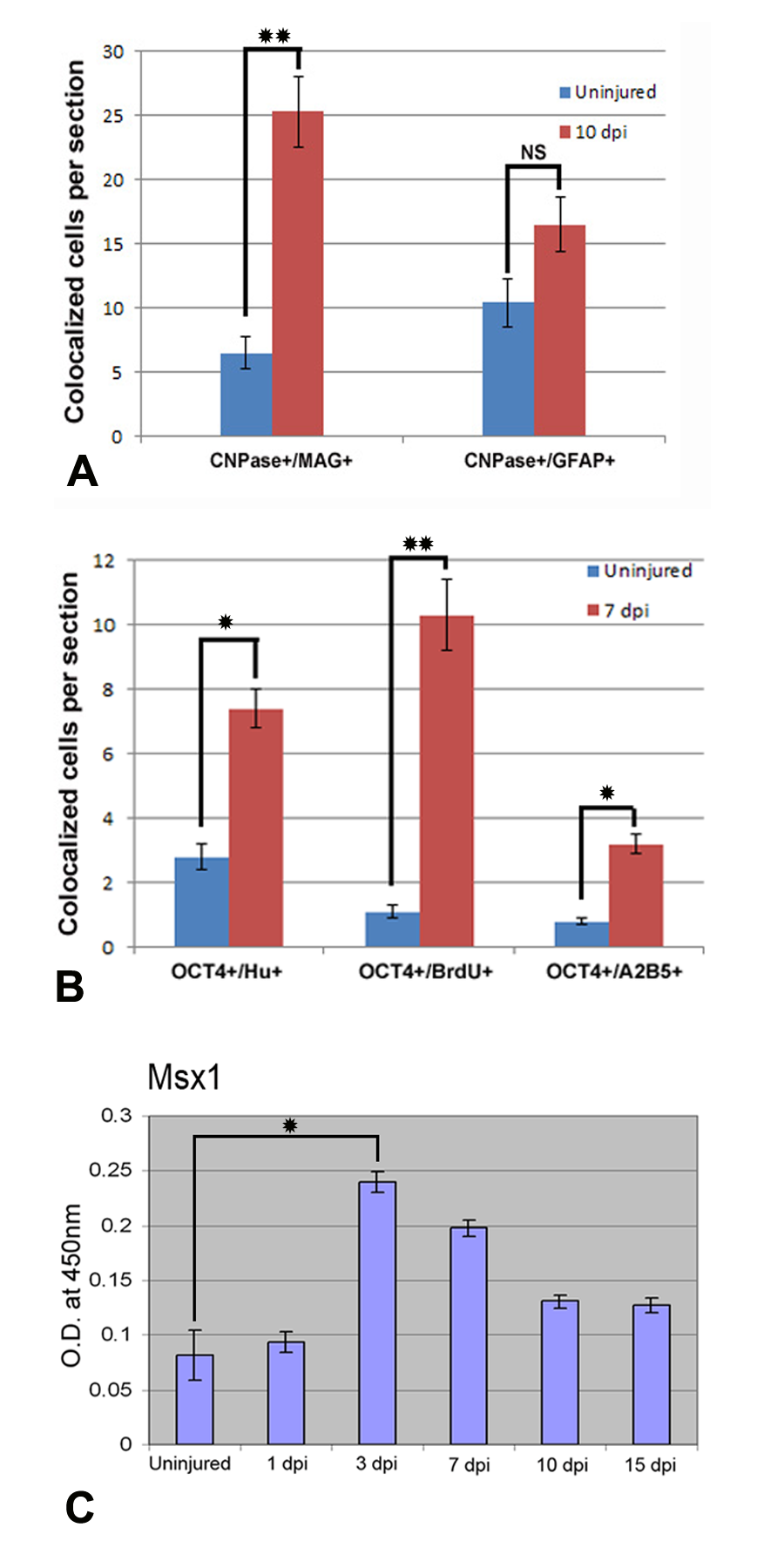

Supplement: S4 Fig — A) Quantification of CNPase+/MAG+ and CNPase+/GFAP+ cells in uninjured and 10 dpi cord. Values represent as mean ± s.e.m. (n = 3). Statistical significance represented as p value (Student’s t-test; **p<0.01, ‘NS’ denotes not significant). B) Quantification of OCT4+/HuC/D+, OCT4+/BrdU+ and OCT4+/A2B5+ colocalized cells in uninjured and 7 dpi cord. Values represent as mean ± s.e.m. (n = 3). Level of significance represented as p value (Student’s t-test; *p<0.05, **p<0.01). C) Quantitative expression of Msx-1 protein at different time points by using ELISA. Values indicate mean ± s.e.m. (n = 3) and statistical significance shown as p value (ANOVA; *p<0.05). (TIF) [file pone.0143595.s004.tif]

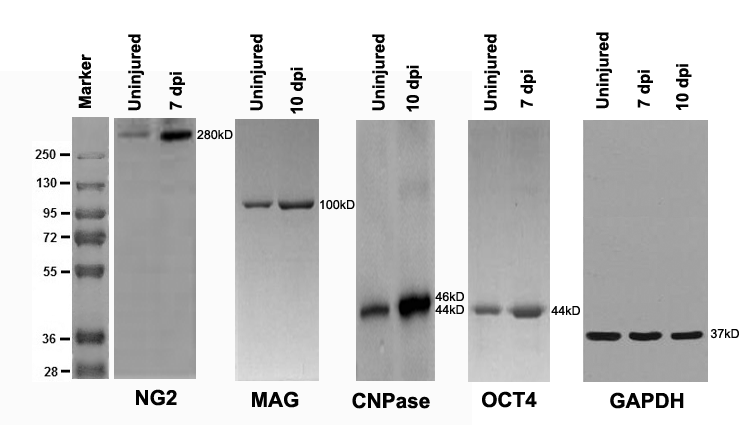

Supplement: S5 Fig — Western blot analysis of MAG, CNPase, NG2 and OCT4 protein in zebrafish uninjured and injured (10 dpi and 7 dpi cord respectively) spinal cord tissues. GAPDH represents as internal loading control for uninjured and respective injured spinal cord tissue. (TIF) [file pone.0143595.s005.tif]

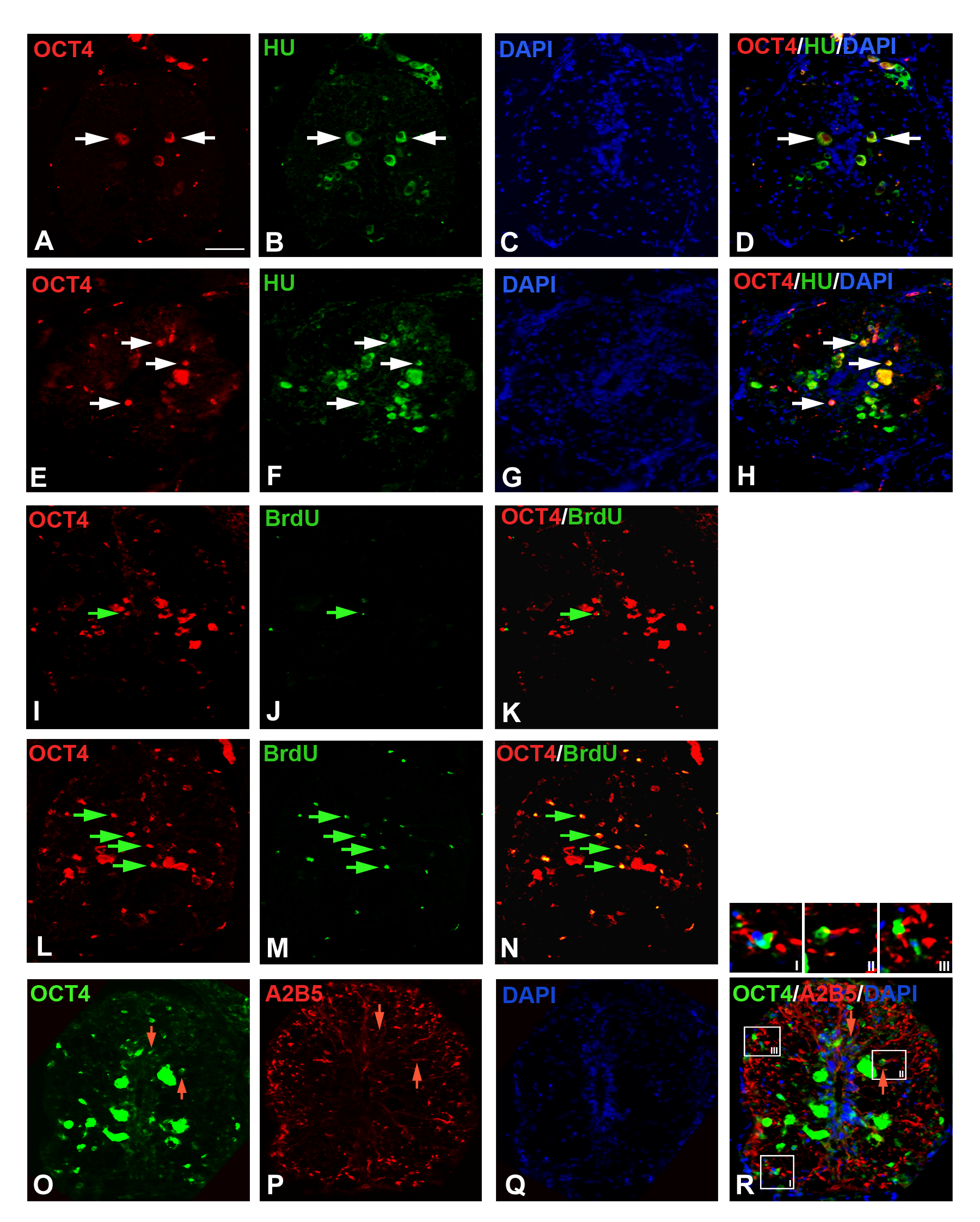

Supplement: S6 Fig — A-D) Uninjured cord section of Fig 5I shown in separate images immunostained with OCT4, HuC/D and DAPI. E-H) The 7 dpi cord section of Fig 5J shown in separate images immunostained with OCT4, HuC/D and DAPI. I-K) Uninjured cord section of Fig 5K shown in separate images immunostained with OCT4 and BrdU. L-N) The 7 dpi cord section of Fig 5L shown in separate images immunostained with OCT4 and BrdU. O-R) The 7 dpi cord section of Fig 5M shown in separate images immunostained with OCT4, A2B5 and DAPI. The insets I, II and III in panel R, indicate three different representative OCT4 and A2B5 colocalized cells. Scale bar = 20 μm. (TIF) [file pone.0143595.s006.tif]
