# Supplementary material for: Characterization of Proliferating Neural Progenitors after Spinal Cord Injury in Adult Zebrafish
Source: PLoS One. 2015 Dec 2;10(12):e0143595. doi: 10.1371/journal.pone.0143595 (PMC4667880; doi:10.1371/journal.pone.0143595)
Supplement: S1 Table — Values represented as mean ± s.e.m. (n = 5), Statistical significance as p value (Student’s t-test; **p<0.01, ***p<0.001). (DOC) [file pone.0143595.s007.doc]

**Supplementary Table 1: Quantification of Sox2^+^ cells colocalized with BrdU, HuC/D and GFAP in uninjured and injured cord at various time points.**

| **Colocalized Sox2^+^ cell types** | **Uninjured**  **(Mean±s.e.m.)** | **3 dpi**  **(Mean±s.e.m.)** | **7 dpi**  **(Mean±s.e.m.)** |
| --- | --- | --- | --- |
| **Percentage of Sox2^+^/BrdU^+^ cells among Sox2^+^ cells** | 1.83±0.28 | 8.34±0.52 ** | 11.98±0.95 *** |
| **Percentage of Sox2^+^/Hu^+^ cells**  **among Sox2^+^ cells** | 0.46±0.28 | 6±0.45 ** | 9.03±0.56 *** |
| **Percentage of Sox2^+^/GFAP^+^ cells**  **among Sox2^+^ cells** | 1.65±0.21 | 5.69±0.40 ** | 5.81±0.63 ** |

P value compared to uninjured cord (**p<0.01, ***p<0.001; n=5)
