# Supplementary material for: Characterization of Proliferating Neural Progenitors after Spinal Cord Injury in Adult Zebrafish
Source: PLoS One. 2015 Dec 2;10(12):e0143595. doi: 10.1371/journal.pone.0143595 (PMC4667880; doi:10.1371/journal.pone.0143595)
Supplement: S2 Table — Values represented as mean ± s.e.m. (n = 5), Statistical significance as p value (Student’s t-test; **p<0.01, ***p<0.001). (DOC) [file pone.0143595.s008.doc]

**Supplementary Table 2: Quantification of A2B5^+^, NG2^+^, A2B5^+^/BrdU^+^ and NG2^+^/BrdU^+^ cells in uninjured and injured cord at various time points.**

| **Cell types** | **Uninjured**  **(Mean±s.e.m.)** | **3 dpi**  **(Mean±s.e.m.)** | **7 dpi**  **(Mean±s.e.m.)** | **15 dpi**  **(Mean±s.e.m.)** |
| --- | --- | --- | --- | --- |
| **Percentage of A2B5^+^ cells among DAPI^+^ cells** | 6.4±0.85 | 4.2±0.62 | 14.3±1.69** | 7.5±1.25 |
| **Percentage of A2B5^+^/BrdU^+^ colocalized cells**  **among A2B5^+^ cells** | 1.1±0.31 | 5.6±0.76 | 16.8±2.20*** | 3.9±0.75 |
| **Percentage of NG2^+^ cells among DAPI^+^ cells** | 3.3±0.57 | 2.4±0.42 | 10.7±1.30** | 4.1±0.67 |
| **Percentage of NG2^+^/BrdU^+^ colocalized cells among NG2^+^ cells** | 0.92±0.25 | 3±0.62 | 7.9±1.13*** | 2.8±0.43 |

Uninjured vs. 7 dpi (**p<0.01, ***p<0.001; n=5)
