# Supplementary material for: Characterization of Proliferating Neural Progenitors after Spinal Cord Injury in Adult Zebrafish
Source: PLoS One. 2015 Dec 2;10(12):e0143595. doi: 10.1371/journal.pone.0143595 (PMC4667880; doi:10.1371/journal.pone.0143595)
Supplement: S3 Table — (DOC) [file pone.0143595.s009.doc]

**Supplementary Table 3: Comparison of pluripotency related genes expressed in various regenerating tissues in different species.**

| **A) Pluripotency related gene expression during Zebrafish fin regeneration (Christen et al., 2010)** | **B) Pluripotency related gene expression during Xenopus limb regeneration (Christen et al., 2010)** | **C) Pluripotency related gene expression during zebrafish spinal cord regeneration (Hui et al., 2014)** |
| --- | --- | --- |
| *pou5f1, sox2, zic3, klf4, c-myc, sall4, tert, mps1/ttk, hsp60, msxb, hsp90a1.* | *oct25, oct60, oct79, oct91, sox2, c-myc, lin28, zic3, tert-A, cripto3, gdf3, sall4, dppa2/4, fut1, msx1, fgf8, lef1.* | *tgfb1, wnt7a, slpr1, prkar1a, sox2, fzd2, wnt4a, wnt4b, mmp9, tgfbr1, smad3a, fgfr1, gsk3b, bmpr2a, smad1, fzd31, tcf712, rac3, pik3cd, fgfr2, smad2, bmpr1b, bmp15, pou5f1, lef1, wnt11r, gdf9, ttk, hsp90ab1, msxc, msxe, vim, stat1b, mycn, stat3.* |
